# Comparative analysis of COVID-19 responses in Japan and Africa: diet, phytochemicals, vitamin D, and gut microbiota in reducing mortality—A systematic review and meta-analysis

**DOI:** 10.3389/fnut.2024.1465324

**Published:** 2024-10-07

**Authors:** Kazuki Santa, Raita Tamaki, Kenji Watanabe, Isao Nagaoka

**Affiliations:** ^1^Faculty of Medical Sciences, Juntendo University, Chiba, Japan; ^2^Department of Biotechnology, Tokyo College of Biotechnology, Tokyo, Japan; ^3^Institute of Tropical Medicine, Nagasaki University, Nagasaki, Japan; ^4^Yokohama University of Pharmacy, Kanagawa, Japan; ^5^Department of Biochemistry and Systems Biomedicine, Graduate School of Medicine, Juntendo University, Tokyo, Japan

**Keywords:** COVID-19, phytochemicals, polyphenols, flavonoids, vitamin D, gut microbiota, Japan, Africa

## Abstract

**Background:**

As the novel coronavirus disease 2019 (COVID-19) pandemic subsides, the clinical sequelae are becoming more problematic. Interestingly, the statistical data indicate that Africa has experienced the lowest number of cases and deaths, with an unexpected phenomenon where the number of deaths from COVID-19 has not increased significantly. Several studies have investigated the relationship between diet and coronavirus. However, no systematic review/meta-analysis has conclusively linked diet (phytochemicals and vitamin D) and the gut microbiota in the context of COVID-19.

**Methods:**

This study examined the responses to COVID-19 in Japan and Africa, formulating the following hypotheses: (1) a healthy diet is effective against COVID-19, (2) blood vitamin D levels are associated with COVID-19 mortality, and (3) COVID-19 is associated with the gut microbiota. To investigate these hypotheses, a keyword search and meta-analysis were conducted using PubMed, and each hypothesis was tested.

**Results:**

This study found that a healthy diet, particularly rich in phytochemicals such as polyphenols and flavonoids, is effective against COVID-19. An association was detected between blood vitamin D levels and COVID-19 mortality. The gut microbiota was linked to COVID-19 and its amelioration. These findings may have significant implications for not only understanding COVID-19 but also future prevention of pneumonia.

## 1 Introduction

The COVID-19 pandemic has caused a global crisis, reminiscent of the Spanish flu of 1918, with severe consequences for the global economy. Pneumonia caused by coronaviruses is a zoonosis, and humans have experienced the emergence of three highly pathogenic CoV species over the past two decades: severe acute respiratory syndrome (SARS)-CoV, Middle East respiratory syndrome (MERS)-CoV, and SARS-CoV-2 (1). Its strong infectivity has been verified through transmission from humans to cats, which may have served as intermediate hosts for the virus (2). Regarding the COVID-19 vaccine, some ecological studies have shown regional disparities in immunization coverage in the USA (3). There is concern regarding low vaccination rates despite the greater risk of infection in non-Hispanic Black and Hispanic populations. Data provided by the WHO as of June 17, 2024, showed that the COVID-19 deaths in Africa, the Americas, Europe, the Eastern Mediterranean region, and Asia (Western Pacific and South-East Asia) numbered 175,510 (2%), 3,020,756 (43%), 2,272,390 (32%), 351,975 (5%), and 1,229,712 (17%), respectively (4). Africa had the lowest proportion of cumulative deaths worldwide at 2%, accounting for 9,579,844 cumulative cases and only 1% of the global total.

Contrary to expectations, the number of COVID-19 deaths did not increase significantly in Africa despite the high rates of HIV, malaria, and other infectious diseases and the lack of developed healthcare systems. In contrast, in many developed countries in Europe and the USA, which have large elderly populations, COVID-19 resulted in high mortality rates, especially among the elderly and those with underlying medical conditions. Elderly individuals are more susceptible to pneumonia, with underlying conditions such as diabetes and obesity, which are metabolic conditions included in lifestyle-related diseases, and a weakened immune system due to various diseases. These susceptible populations prioritized vaccination and other preventive measures.

In Africa, an interesting phenomenon was observed: the number of deaths due to COVID-19 did not increase, as expected. However, this anomaly requires further investigation. One possible reason for this is the demographic structure of Africa, which has an overwhelmingly large number of children and a relatively small number of elderly individuals, who showed higher mortality rates from COVID-19. The median age on the African continent is 18.8 years, compared to the world average of 30.7 years and 49.5 years in Japan (5). In children, the innate immune response that eliminates the virus may be more effective than the speed at which the virus mutates (6, 7).

Another explanation is that Japan’s low mortality rate from COVID-19 compared to that in Western countries comes from its status as the country with the longest life expectancy in the world (8). Reports have shown that the nutritional situation in Africa has been adversely affected by the COVID-19 pandemic, particularly among children (9). In addition to economic development, a well-known factor contributing to Japan’s longevity is the healthiness of the Japanese diet. The Japanese diet, similar to the Mediterranean diet (10), has also been considered healthy, particularly around 1975, which is considered healthier than the current Japanese diet (11–13). Compared to the USA and other countries, Japan has lower rates of obesity and lifestyle-related diseases, including metabolic diseases, which are believed to be associated with the longevity of its population. The typical Western diet is high in energy density, leading to underlying and lifestyle-related diseases and impaired immunity due to chronic inflammation, which have been identified as risk factors for COVID-19 (14). This study investigated the effects of polyphenols, a class of phytochemicals abundant in the healthy diets of the Mediterranean region and Japan, on COVID-19.

It is also well-established that vitamin D deficiency is associated with a range of diseases, including those that impair immunity. A systematic review and meta-analysis conducted in Italy, a country severely affected by COVID-19, revealed a clear association between vitamin D deficiency and COVID-19 mortality (15). Blood vitamin D levels are categorized as follows: < 20 ng/mL deficient, 20–30 ng/mL, insufficient; and > 30 ng/mL, sufficient. A report showed the following blood vitamin D (25-OH-D) levels (ng/mL) in European countries: France 24.0, Germany 20.0, Italy 20.0, the UK 19.0, and Spain 17.0 (16). In Japan, only 2% of individuals have sufficient blood vitamin D levels, with an average value of 15.5 ng/mL (17). In Africa, because of the strong direct sunlight, over 40% of people have blood vitamin D levels above 30 ng/mL, with a mean value of 27.1 ng/mL, the highest among the compared regions (18).

Finally, the relationship between the gut microbiota and COVID-19, which is significantly influenced by diet and varies with the disease, was also examined.

Based on the above, this study tested the following hypotheses by examining COVID-19 responses in Japan and Africa: (1) a healthy diet is effective against COVID-19, (2) blood vitamin D levels are associated with COVID-19 mortality, and (3) COVID-19 is associated with the gut microbiota. To test these hypotheses, we conducted a series of literature searches and summarized the findings of a systematic review and meta-analysis.

## 2 Materials and methods

### 2.1 Selection criteria, sources, and search strategy

This systematic review follows Cochrane guidelines and reports using Preferred Reporting Items for Systematic Reviews and Meta-Analyses (PRISMA) (19). Compliance with the 2020 Preferred Reporting Items for Systematic Reviews and Meta-Analyses checklist is shown in the Supplementary Table S1. Articles with specific keywords in the title or abstract were selected for this study. The PubMed search engine was used for this systematic review and meta-analysis. The keywords used in this systematic review are: Japan, Africa, polyphenol, flavonoids, vitamin D, and gut microbiota. Polyphenols and flavonoids are major classes of phytochemicals. Four patterns of keyword searches have been shown in the results: Analysis Group A–D.

The protocol was registered in UMIN-CTR under the UMIN study ID: UMIN000054334.^1^ Ethical approval was not required for this study, as all the data used are publicly available. The examined literature was peer-reviewed and written in English. The search for COVID-19-related articles covered the period from 2019 to the 25th of July 2024.

### 2.2 Selection procedure and exclusion criteria

Records were initially identified using a PubMed database search. Duplicate records were excluded from the systematic review. Two independent reviewers (KS and RT) assessed the titles, abstracts, and entire articles, including the results of the identified studies, and judged the inclusion and exclusion of any irrelevant reports. Disagreements regarding the inclusion of studies were resolved through discussions and consensus. If disagreements persisted, they were arbitrated by another reviewer. One example of an excluded study is changes in blood 25-OH-D concentration, bone markers, and physical performance due to vitamin D supplementation while COVID (COVID-19) lock down since this study might appear to meet the inclusion criteria, but these were excluded because they were not related to COVID-19 treatment (20). In some analysis groups, articles other than randomized clinical trials (RCTs) were also excluded. A summary of information derived from up-to-date studies on vitamin D was also used as background information.

### 2.3 Data items and data collection process

Data collection included the following elements: study characteristics (author, year of publication, title, and abstract), participants (selected from relevant RCTs in the context of COVID-19 or long-COVID-19) and keywords in each grouped analysis.

**Analysis group A**: A two-keyword search was conducted to summarize the COVID-19 responses in Japan and Africa. A keyword search (Japan), (COVID), and (vitamin D, polyphenol, flavonoids, or gut microbiota) yielded 100 results. Only one study was an RCT. Therefore, top 10 search results sorted according to “Best Match” and content related to COVID-19 were showed in the results. Only articles where the full text was available for free were used. Another keyword search, (Africa) and (COVID) and (vitamin D or polyphenol or flavonoids or gut microbiota) yielded 53 results, among which 40 had the full text available for free. Then, top 10 search results sorted by “Best Mach” and title related with COVID-19 were showed in the Tables 1, 2 in the results. Furthermore, two of the four RCTs in the search results as well.

**Analysis Group B**: For the blood 25-OH-D and COVID-19, search results for (COVID) and (vitamin D) were classified as enough (> 30 ng/mL), insufficient (20 to 30 ng/mL), and deficient (< 20 ng/mL) by the mean concentration of the intervention groups. Of the 1,893 results, 48 were RCTs and were further filtered out based on the availability of free full texts, resulting in 43 articles. From these results, 20 articles containing values of blood 25-OH-D3 with mean ± SD or median were summarized in the table. In addition, a meta-analysis was conducted using the data contained in these articles. Furthermore, a meta-analysis of enough blood 25-OH-D levels (> 30 ng/mL) in the intervention group was conducted.

**Table 1:** Analysis Group A: Top 10 articles sorted by best match in PubMed - Japan, COVID and (polyphenol or flavonoids or vitamin D or gut microbiota) (As of 25th of July).

Research	Main findings	First author, year, references
Fundamental immuno modulatory effects of vitamin D in COVID-19 pandemic (Review)	Anti-inflammatory effects of 1α-25-(OH)2-D through VRDs and 1α-hydroxylase expressed on the immune cells in COVID.	Ao et al. (2021) (23)
Fecal shotgun metagenomic sequencing and metabolomics in SARS-CoV-2 infected 112 hospitalized patients and 112 control subjects.	Discovery of correlations between oral microbes, short-chain fatty acid producers, and intestinal metabolites associated with COVID-related microbes, and association with inflammatory cytokine dynamics.	Nagata et al. (2023) (24)
Summary of intestinal barrier (mechanical, chemical, microbial, and immune barrier) disruption mechanism by SARS-CoV-2 (Review).	Presentation of disruptive mechanisms of intestinal integrity of mechanical, chemical, microbial, and immune barriers by SARS-CoV-2 infection in COVID-19 including gastrointestinal symptoms.	Xue et al. (2023) (25)
Association between trypsin self-degradation by Paraprevotella strains and severity of diarrhea in COVID-19 patients.	Colonization of *Paraprevotella* strains inhibits the mouse coronavirus lethal infection through the inhibition of trypsin and trypsin-like protease dependent host cell invasion.	Li et al. (2022) (26)
Effects of tea catechins in SARS-CoV-2 Omicron subvariant.	Green tea, Matcha and black tea polyphenols effectively inactivate SARS-CoV-2 Omicron subvariant.	Shin-Ya et al. (2023) (27)
Research in oral fluid-based biomarkers in the detection of SARS-CoV-2 in saliva.	Oral and periodontal disease biosensor and lab-on-a-chip biomarkers detect SARS-CoV-2 in saliva.	Steigmann et al. (2020) (121)
Anti-viral (COVID) and anti-inflammatory effects of phytochemical-containing essential oil (Review).	Olfactory training with phytochemicals contained in lemon, rose, clove, and eucalyptus essential oil improve olfactory functions.	Koyama et al. (122)
Changes of microbiome in COVID-19 (Clinical study).	Gut microbiota diversity increase after the recovery from COVID-19, protective effects of Bacteroids in severe SARS-CoV-2 infection.	Babszky et al. (123)
Improvement of oxidative stress in COVID-19 outpatients by vitamin D supplementation.	Comparison between COVID patients and healthy subjects in anti-oxidative and anti-inflammatory effects, vitamin D supplementation suppress SOD, GPx, and TAC levels in COVID patients.	Golabi et al. (2022) (124)
High body temperature induced by the influenza A virus and SARS-CoV-2 infection increases gut microbiota-dependent host resistance.	Physiological role of fever in host resistance to viral infection, upregulation of immune response. Gut microbiota produced deoxycholic acid (DCA) and TGR5 signaling pathways suppress the viral replication and neutrophil dependent tissue damage.	Nagai et al. (2023) (125)

**Table 2:** Analysis Group A: Top 10 articles sorted by best match in PubMed - Africa, COVID and (polyphenol or flavonoids or vitamin D or gut microbiota) (As of 25th of July).

Research	Main findings	First author, year, references
The early age and plant-based diet hypotheses of low SARS-CoV-2 infection and the COVID-19 pandemic in sub-Saharan Africa (review)	Higher metabolic syndrome ratio is associated with higher risk of COVID infection. Africa has the lowest ration of metabolic syndrome. Plant-based diet includes whole grain, legumes, vegetables, potatoes, pumpkins, banana, moringa leaves, and reduced meat consumption. Plant based diet provides unique gut microbiome and extended survival ratio.	Losso et al. (2021) (28)
Acute and subacute oral toxicity characterization and safety assessment of Madagascar’s anti-COVID herbal tea in animal models.	Herb tea consists of *Artemisia annua* (62%), and other plants (38%) was confirmed safe in mice.	Aina et al. (2023) (126)
Comparative analysis of Beninese and Chinese herbal medicine in COVID-19 treatment.	Identified herbal medicine used in Benin compared with Chinese herbal medicine, efficacy was vitrified *in vitro*. *Citrus aurantiifolia* (13.18%), *Momordica charantiantia* (7.75%), *Ocimum gratissimum* (7.36%), *Crateva adansonii* (6.59%), *Azadirachta indica* (5.81%), *Zanthoxylum zanthoxyloides* (5.42%) were the most used.	Houeze et al. (2023) (29)
Efficacy of propolis in SARS-COV-2 virus: anti-viral effects and molecular simulation (Review)	Propolis polyphenol reduce the replication of virus and beneficial for the treatment of SARS-CoV-2 infected patients.	Ghosh et al. (2022) (127)
Effects of resveratrol in COVID-19 (Review)	Resveratrol in safe, affordable, and available adjuvant treatments.	van Brummelen et al. (2022) (30)
Randomized trials, meta-epidemiological cohort study of hydroxychloroquine, corticosteroids, and vitamin D in COVID-19 (meta-analysis).	Hydroxychloroquine, corticosteroids, and vitamin D as a treatment of COVID-19, less than one third of registered trials made their results public.	Fincham et al. (2024) (31)
Research of effective molecular against coronavirus protease using flavonoids.	Inhibition of SARS-CoV-2 main protease (Mpro) by quercetin-3-O-Neohesperidoside is the candidate of COVID-19 treatment.	Fadaka et al. (2020) (128)
Research of COVID-19 severity and vitamin D levels.	COVID-19 patients in 82% were vitamin D deficiency or insufficient. Patients in vitamin D deficiency were higher risk of COVID-19 infection.	Kalichuran et al. (2022) (32)
Verification of cytotoxic and anti-viral effects of *Bersama abyssinica* extract in SARS-CoV-2 delta variant.	*B. abyssinica* water extract used in COVID-19 treatment had significant antiviral effect in SARS-CoV-2 but no cytotoxic in Vero E6 cells.	Zekeya et al. (2022) (129)
Effects of probiotics in the war against COVID virus (Review).	Intestinal probiotics, lactic acid bacteria (LAB) and *Bifidobacterium* spp. were decreased in COVID-19 patients. Explored the potential of probiotic bacteria and their metabolites to intervene with the process of virus infection.	Tiwari et al. (2020) (130)

**Figure 1:**
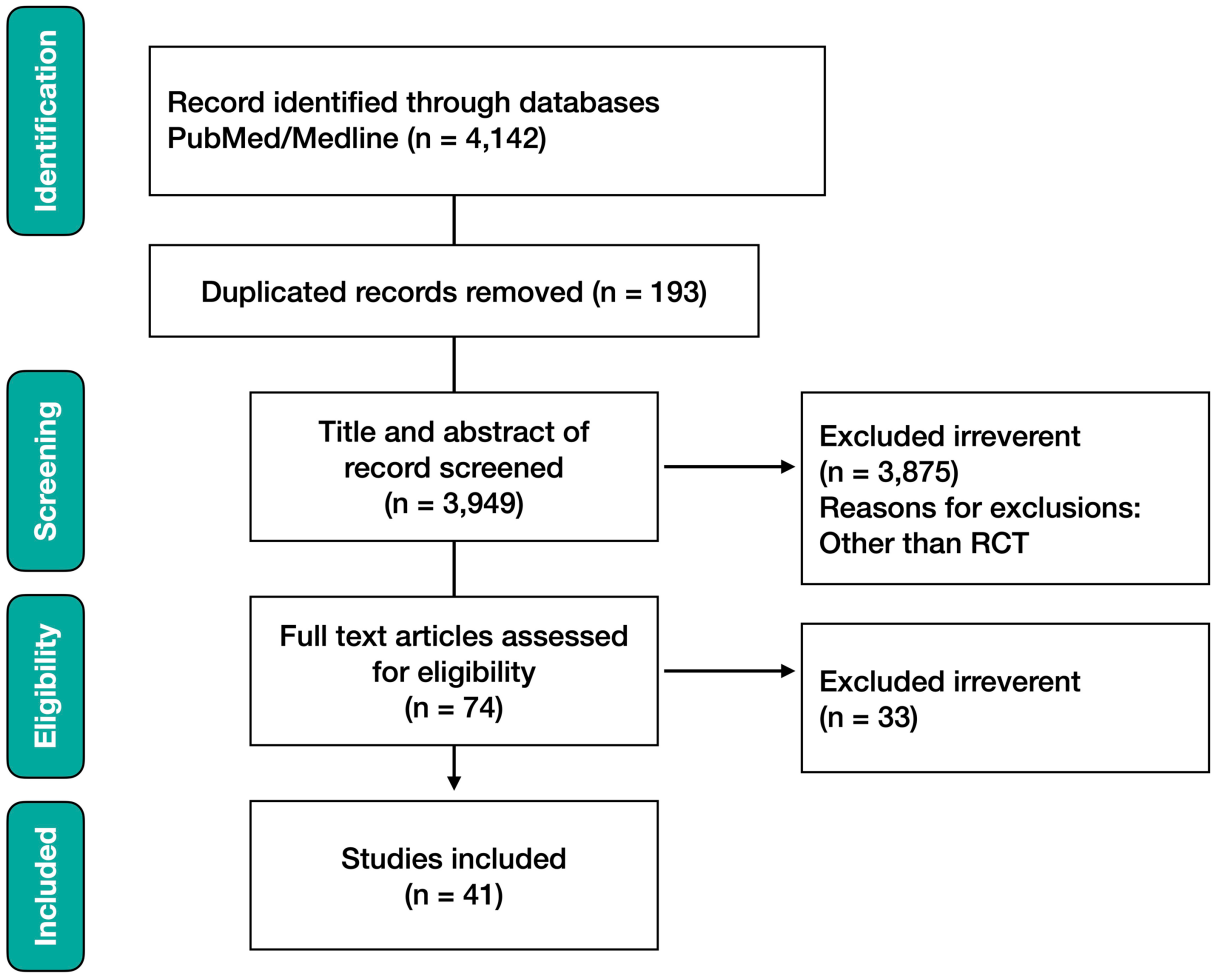
PRISMA flowchart of this systematic review: Analysis Group A. PubMed search with (COVID) and each keyword (polyphenol or flavonoids), (vitamin D), and (gut microbiota) showed 4,142 results. Combined search of (COVID) and (polyphenol or flavonoids or vitamin d or gut microbiota) showed 3,949 results. Duplicated 193 results were excluded. Then, 3,875 results other than randomized clinical trials (RCTs) were excluded and only 74 RCTs were considered further analysis. Of these, 33 studies were excluded based on the exclusion criteria because they did not meet the purpose of the analysis. Finally, 41 manuscripts were exploited in this systematic review (as of 25th of July 2024).

**Analysis group C**: Combined keywords (COVID and polyphenol) yielded 410 results, (COVID and flavonoids) yielded 818 results, (COVID and vitamin D) yielded 1,893 results, and (COVID and gut microbiota) yielded 1,012 results. A total of 4,142 studies appeared in the PubMed search. A combined search of (COVID) and (polyphenols, flavonoids, vitamin D, and gut microbiota) showed 3,949 results. Therefore, 193 duplicate results were excluded. As only RCTs were considered, 3,875 articles were excluded, leaving 74 eligible papers. Of these, 33 studies were excluded because they did not meet the exclusion criteria described above. Finally, 41 studies were included in this systematic review. The screening process is described in detail in the PRISMA flowchart (Figure 1). A meta-analysis was performed on the search results for (COVID and vitamin D) in the analysis group C.

**Analysis group D**: Existing articles within the 5 years when COVID-19 related articles were available were searched for in PubMed. A combination of keywords (polyphenols or flavonoids) and (gut microbiota) yielded 2,979 results. Within the last 5 years when COVID-19 was prevalent, 2,225 search results were obtained. Of these, 48 were RCTs. When narrowed down to the past year, 573 papers and 9 RCTs were identified, fitting the study’s objectives, resulting in seven papers being included. A PubMed search for (vitamin D) and (gut microbiota) yielded 532 results, with 389 results published in the last 5 years. Among these, 16 were RCTs, and five were fit-for-purpose articles. A PubMed search for (polyphenols, flavonoids) and (vitamin D) yielded 622 results, of which 188 were obtained in the last 5 years. Of these, 11 were RCTs, and six were fit-for-purpose articles.

### 2.4 Outcome

To verify the three hypotheses: (1) a healthy diet is effective against COVID-19, (2) blood vitamin D levels are associated with COVID-19 mortality, and (3) COVID-19 and gut microbiota are associated, search results from Analysis Groups A, B, C, and D have been summarized into tables in the results section.

### 2.5 Statistical analysis

A series of meta-analyses of the articles corrected in Analysis Groups B (COVID and vitamin D) and C were conducted (articles in Tables 3–5, 8). Meta-analysis has been performed by EZR [R 4.4.1 binary for macOS 11 (Big Sur)] software downloaded from “The Comprehensive R Archive Network” webpage.^2^ References including median values were converted to mean ± SD from the first and third tetrad counts. Meta-analysis for means were conducted to analyze the data sets including mean ± SD and total number of samples. The standard mean difference (SMD) with 95% confidence interval (CI) was reported for dichotomous outcomes. A meta-analysis for proportions was conducted to analyze the datasets, including events and the total number of samples. Odds ratios (ORs) with 95% confidence intervals (CI) were reported for dichotomous outcomes. This study performed both fixed-and random-effects modeling. *p* < 0.01 was considered statistically significant.

**Table 3:** Analysis Group B: Association between COVID-19 and blood 25-OH-D3 levels; mean value of the Intervention group showed enough level (> 30 ng/mL) RCT.

First author, year, references	Subjects numbers		Mean 25-OH-D (ng/mL)		SD	
	Intervention	Control	Intervention	Control	Intervention	Control
Mariani et al. (2022) (131)	115	103	102.00	30.00	34.81	2.59
Bishop et al. (2023) (132)	65	69	82.00	37.00	4.00	1.00
Fernandes et al. (2022) (133)	101	99	44.60	19.80	14.70	10.50
Murai et al. (2021) (134)	120	120	44.40	19.80	15.00	10.50
Jolliffe et al. (2022) (135)	956	908	42.16	21.44	9.40	10.08
Mahjoub et al. (2024) (136)	34	24	42.00	19.30	13.70	8.50
Haas et al. (2024) (137)	17	41	35.36	37.20	11.04	12.24
Karonova et al. (2022) (50)	45	46	32.90	19.30	9.85	9.70
Murai et al. (2021) (138)	16	16	31.70	7.80	12.30	1.70
Caballero-García et al. (2021) (139)	15	15	31.30	19.40	1.40	2.30

**Table 4:** Analysis Group B: Association between COVID-19 and blood 25-OH-D3 levels; mean value of the Intervention group was sufficient level (20 to 30 ng/mL) RCT.

First author, year, references	Subjects numbers		Mean 25-OH-D (ng/mL)		SD	
	Intervention	Control	Intervention	Control	Intervention	Control
Brunvoll et al. (2022) (140)	278	17,323	29.64	25.12	8.27	9.90
Torres et al. (2022) (52)	41	44	29.22	19.11	6.89	8.69
Cannata-Andía et al. (2022) (141)	274	269	29.00	16.40	10.89	7.78
Cesur et al. (2023) (48)	16	17	27.18	14.78	12.08	10.75
Villasis-Keever et al. (2022) (142)	94	98	26.10	19.30	7.41	8.52
Sabico et al. (2021) (53)	36	33	25.00	23.96	1.36	1.56
Karonova et al. (2022) (143)	56	54	22.80	10.60	7.04	4.81
Annweiler et al. (2022) (47)	127	127	21.20	17.20	12.15	17.19
Bychinin et al. (2022) (51)	55	55	20.60	9.60	9.63	11.11

**Table 5:** Analysis Group B: Association between COVID-19 and blood 25-OH-D3 levels; mean value of the Intervention group was insufficient level (< 20 ng/mL) RCT.

First author, year, references	Subjects numbers		Mean 25-OH-D (ng/mL)		SD	
	Intervention	Control	Intervention	Control	Intervention	Control
De Niet et al. (2022) (144)	50	50	17.87	16.87	0.15	9.48

## 3 Results

### 3.1 Comparison of Japan and Africa’s population

**Figure 2:**
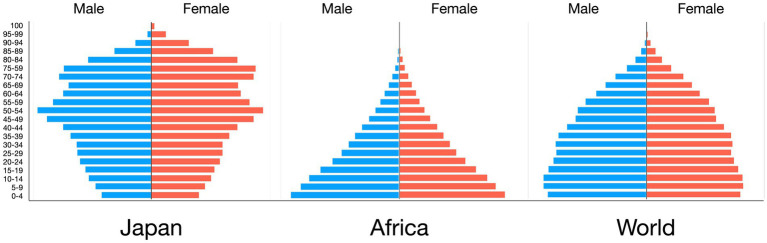
Population pyramid of Japan, Africa, and the World in 2024. Population pyramid of Japan was a typical shape of an aged society with large numbers of baby boomer seen in developed countries. Population groups peaked around 45–49 and 70–74 years old. Africa had a typical juvenile-formed population pyramid. Population pyramid of the world total was bell-shaped. One reason of Africa’s low death ratio of COVID-19 seemed to come from this very young population distribution.

Figure 2 shows the population pyramids of Japan, Africa, and the rest of the world. The largest population group in Japan peaked in the age group of 50–54 (9,510,374 people), followed by the age group of 70–74 (8,218,437 people). These were the second and first baby boomers, respectively (21). The Japanese population is a typical example of an aged society that is common in developed countries, with people older than 65 years comprising a quarter of the population. Surprisingly, the proportion of women older than 100 years was 0.1% in Japan. In contrast, Africa had a typical juvenile population pyramid; as the population became younger, the number of people in Africa increased. However, the world population showed a bell-shaped pyramid. Since COVID-19 vaccination efforts were prioritized for the elderly and people with underlying diseases in Japan, an African population with an enormous number of children was considered one of the reasons for Africa’s low number of COVID-19 deaths.

### 3.2 Analysis group A: Summary of COVID response in Japan and Africa

#### 3.2.1 Summary of COVID response in Japan

Only one RCT was found after searching for Japanese COVID-19 responses to oral vaccination against Tuberculosis (22). Table 1 summarizes the 10 articles on Japan’s response to COVID-19. These articles were publication types other than RCTs, and were conducted in Japan or by authors belonging to Japanese research institutions. They include a review of the effects of vitamin D against COVID-19 through the vitamin D receptors (VDRs) expressed on the surface of immune cells (23); a metabolomics research related to COVID (24); a review of the mechanism of severe gastrointestinal conditions in COVID-19 (25); association between the enhancement of trypsin self-degradation by *Paraprevotella* colonization and severity of diarrhea in patients infected with SARS-CoV-2 (26), and effects of green tea catechins on Omicron variants (27).

#### 3.2.2 Summary of COVID response in Africa

Table 2 summarizes the 10 articles associated with COVID-19 responses in Africa. A review article on plant-based diets in sub-Saharan Africa reasoned that a low metabolic syndrome ratio is associated with a low COVID-19 risk in Africa (28). Others include the identification of herbal plants administrated to COVID patients in Benin (29); resveratrol as a safe, affordable, and available adjuvant treatment (30); meta-analysis of corticosteroids, hydroxychloroquine, and vitamin D as treatments for COVID-19 (31); and a report on high COVID-19 risks in patients with vitamin D deficiency (32). Only four RCTs were found in African research; however, one was associated with tuberculosis prevention (33) and the others were about egg consumption to improve diet (34).

### 3.3 Analysis Group B: Association between COVID-19 and blood 25-OH-D3 levels; deficient, insufficient, and enough amount

Of the 43 RCTs on COVID and vitamin D, 20 articles held mean ± SD or median values were summarized and divided by the serum vitamin D levels of the intervention group into three groups: enough (> 30 ng/mL) amount (Table 3), insufficient (20 to 30 ng/mL) (Table 4), and deficient (< 20 ng/mL) (Table 5). The mean value in the enough amount group varied from 31.3 to 102 (ng/mL), that in the insufficient group varied from 20.8 to 29.64, and the deficiency group had one result with a level of 17.87.

**Figure 3:**
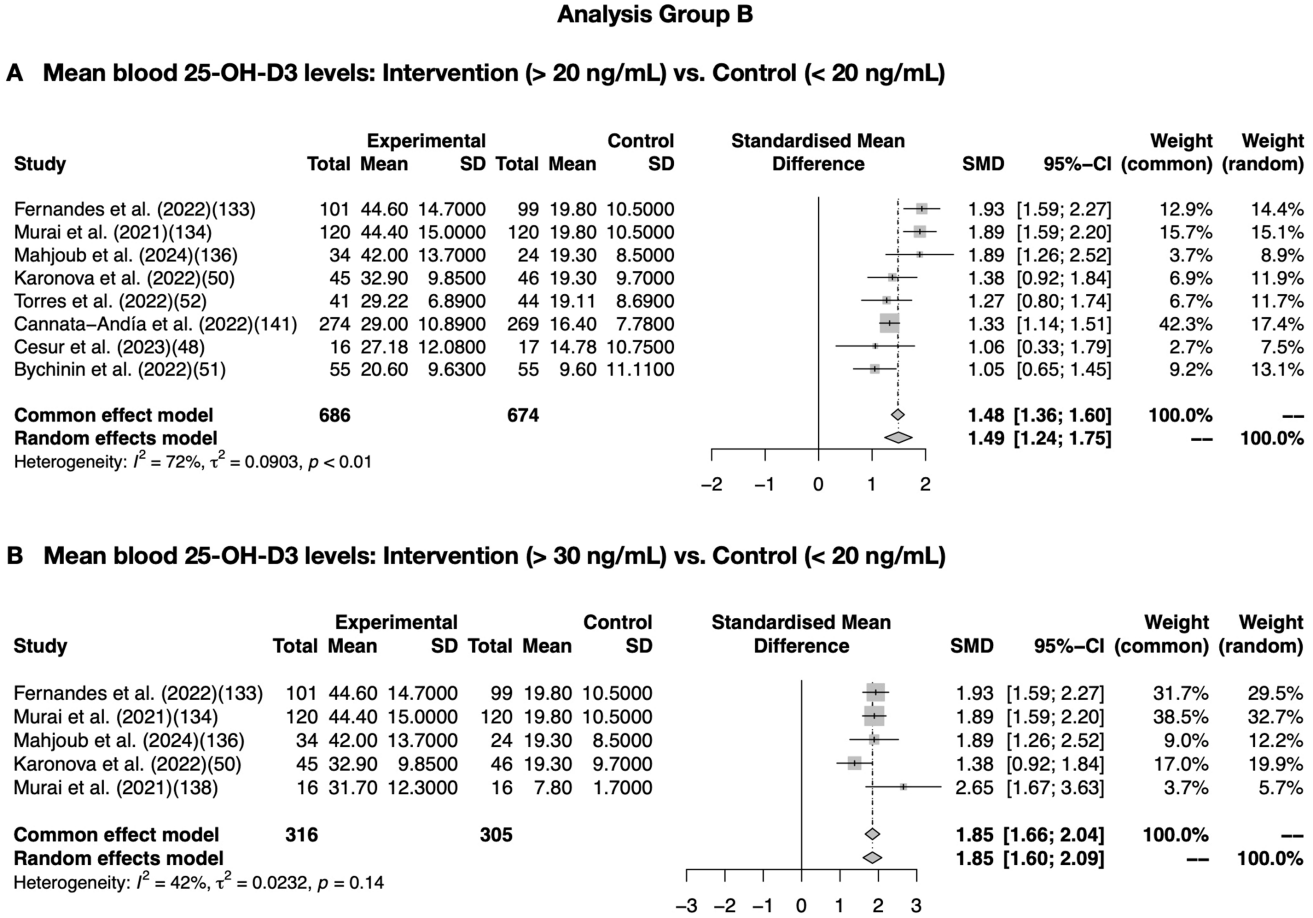
Meta-analysis of association between COVID-19 and vitamin D supplementation in Analysis Group *C. meta*-analysis conducted using data extracted from articles in Tables 3–5. Meta-analysis was performed only on studies that met both conditions: blood vitamin D levels of 20 (ng/mL) or higher in the intervention group and 20 or lower in the control group. **(A)** Showed a meta-analysis <*I*^2^ = 75% of blood 25-OH-D levels extracted from articles in Tables 3–5. **(B)** Further shows meta-analysis in combinations of the data of enough (> 30 ng/mL) blood 25-OH-D levels resulted in *I*^2^ = 0%. *p* < 0.01 was considered as a significant difference. *p* < 0.01 were considered as statistical significance.

Then, meta-analysis was performed only on studies that met both conditions: blood vitamin D levels of 20 (ng/mL) or higher in the intervention group and 20 or lower in the control group. A meta-analysis performed on articles in Tables 3–5, and the results of adequate heterogeneity (I^2^ = 72%) are shown in Figure 3A. Another meta-analysis with enough (> 30 ng/mL) blood 25-OH-D levels in the intervention groups are shown in Figure 3B.

### 3.4 Analysis group C: Nutrients and gut microbiota against COVID-19

#### 3.4.1 Validation of the effectiveness of a healthy diet against COVID-19

This systematic review shows the relevance of polyphenols and flavonoids, two of the most common phytochemicals associated with a healthy diet, in relation to COVID-19. Table 6 summarizes the four RCTs on COVID-19 and polyphenols, and Table 7 summarizes the eight RCTs on COVID-19 and flavonoids. Flavonoids are a typical component of polyphenols. The polyphenol curcumin promotes recovery from COVID-19 by improving blood oxygen saturation (35). Additionally, daily consumption of high-polyphenol olive oil was found to significantly reduce treatment duration (36). Two RCTs involving resveratrol, a polyphenol that was first highlighted for its presence in red wine, were included. The first study demonstrated its effectiveness against respiratory infections, including COVID-19 (37), and the second showed that resveratrol reduced the expression of ACE2, a receptor for COVID-19, in the adipose tissue (38). The flavonoid quercetin was found to reduce the expression of markers associated with COVID-19 severity when combined with anti-viral drugs used to treat COVID-19, such as remdesivir and favipiravir. This included effectively lowering levels of serum alkaline phosphatase (ALP), quantitative C-reactive protein (q-CRT), and lactate dehydrogenase (LDH) (39). Silymarin also reduced alanine aminotransferase levels (40). Several studies in Italy reported that luteolin is effective against olfactory abnormalities, one of the symptoms of COVID (41–45). Additionally, the use of gargles containing the bioflavonoids β-cyclodextrin and Citrox (CDCM) was shown to reduce coronavirus presence (46). Phytochemicals are considered the seventh most abundant nutrient and have been shown to be effective against COVID-19.

**Table 6:** Analysis Group C: Effectiveness of a healthy diet against COVID-19 (COVID and polyphenol) RCT.

First author, year, references	Country	Treatment	Subjects	Main findings	Outcome
Ahmadi et al. (2023) (35)	Iran	Curcumin	Intervention (*n* = 29), Control (*n* = 39), four times/day, 2 weeks	Curcumin with standard COVID-19 treatment enhanced anti-inflammatory effects and reduced the recovery time in mild-to-moderate hospitalized patients	Curcumin improves the time and demand of oxygen therapy and blood Oxygen saturation levels.
Rodríguez-Argente et al. (2023) (36)	Spain	High polyphenolic olive oil	Intervention (*n* = 44), Control (*n* = 40) two times/day (2 mL), 3 months	Reduced median recovery time in high polyphonic olive oil intervention, (3 days vs. 7 days)	Daily high polyphenol olive oil significantly reduces the time of recovery.
McCreary et al. (2022) (37)	USA	Resveratrol	Intervention (*n* = 50), Control (*n* = 50), 3 weeks	Phase 2 study with resveratrol vs. control: Hospitalization (2 vs. 6%), COVID-19 related ER visits (8 vs. 14%)	Resveratrol is effective in the therapy and other respiratory infectious viruses (influenza, Respiratory Syncytial Virus, and Human Rhinovirus).
de Ligt et al. (2021) (38)	Netherlands	Resveratrol	Crossover trial, Obese male, (*n* = 11), 30 days	Resveratrol significantly reduces ACE2 (−40%) and leptin (−40%)	Resveratrol reduces ACE2 expression in adipose tissue.

**Table 7:** Analysis Group C: Effectiveness of a healthy diet against COVID-19 (COVID and flavonoids) RCT.

First author, year, references	Country	Treatment	Subjects	Main findings	Outcome
Shohan et al. (2022) (39)	Iran	Quercetin	Intervention (*n* = 30), Control (*n* = 30), 7 days	Quercetin to Remdesivir or Favipiravir treatment significantly reduce hospitalized period, serum ALP, q-CRT, LDH	Quercetin effectively reduced COVID-19 markers (serum ALP, q-CRP, LDH) in severe cases.
Aryan et al. (2022) (40)	Iran	Silymarin	Intervention (*n* = 25), Control (*n* = 25), 3 times/d, 2 weeks	Significant reduction of alanine aminotransferase (*p* < 0.001)	Recommendation of further clinical trials.
Versace et al. (2023) (41)	Italy	Luteolin	Intervention (*n* = 17), Control (*n* = 17), 8 weeks	Palmitoylethanolamide (PEA)-LUT restores GABAB neurotransmission and cortical plasticity.	PEA-LUT recovers cognitive problems in long-COVID associated disorder patients.
Di Stadio et al. (2023) (42)	Italy	Luteolin	Training + Control (*n* = 38), PEA-LUT 1 times/d (*n* = 48), PEA-LUT 2 times/d (*n* = 40), Training + PEA-LUT (*n* = 76), 90 days	PEA-LUT significantly improve olfactory perception in long-COVID patients (*p* < 0.0001)	Olfactory training and PEA-LUT combined recovers over 6 months of olfactory perception disorders in long-OVID patients.
De Luca et al. (2022) (43)	Italy	Luteolin	(*n* = 69: Female 43: Male 26), 3 months	Subjects in 37.7% (*n* = 26) had mental clouding but severity decreased after 3 months (*p* = 0.02)	PEA-LUT and olfactory training improve memory function in long-COVID associated and chronic olfactory loss.
Di Stadio et al. (2022) (44)	Italy	Luteolin	Intervention (*n* = 130), Control (*n* = 55), 90 days	Improvement of olfactory disorders in intervention (92 vs. 43%)	Combined PEA-LUT with olfactory training improve more individuals with long-COVID associated olfactory disorders than only olfactory trained individuals.
D’Ascanio et al. (2021) (45)	Italy	Luteolin	Intervention (*n* = 7), Control (*n* = 5), 30 days	Significant improvement in olfactory threshold, discrimination, and identification score (*p* = 0.01)	Combination of PEA-LUT and rehabilitation are associated with the improvement of olfactory functions, especially in significant in patients with long olfactory disorders.
Carrouel et al. (2021) (46)	France	β-cyclodextrin and citrox (bioflavonoids) (CDCM)	Intervention (*n* = 88), Control (*n* = 88), 7 days	Significant decrease of SARS-Cov-2 in saliva after 4 h of first CDCM use (*p* = 0.036), effects continued after 7 days.	Daily use of mouthwash holding CDMC reduces viral load in saliva.

#### 3.4.2 Validation of the association between blood vitamin D levels and COVID-19 mortality

Table 8 summarizes the relevant RCTs on COVID and vitamin D. Among the 41 studies, 20 were found to be relevant (1 μg = 40 IU). The studies mainly involved the administration of high concentrations of oral vitamin D3, active calcitriol (1α,25-(OH)2-D3), alfacalcidol, and calcidiol (25-OH-D3), which is used to measure vitamin D levels in the blood. The conversion of 25-OH-D3 to calcitriol is facilitated by enzymes in the kidneys or immune cells. High-dose vitamin D3 has been reported to reduce mortality in COVID-19 patients (47) increase vaccine antibody production (48), suppress cytokine storms (49), increase blood 25-OH-D3 levels (50) and lymphocyte counts (51), shorten hospital stays (52), and recovery time (53), reduce healthcare utilization due to COVID-19 (54). There is substantial evidence that vitamin D supplementation is effective during the COVID-19 pandemic and helps improve sequelae such as loss of taste (55).

**Table 8:** Analysis Group C: Association between blood vitamin D levels and COVID-19 mortality (COVID and vitamin D) RCT.

First author, year, references	Country	Treatment	Subjects	Main findings	Outcome
Annweiler et al. (2022) (47)	France	Single oral high dose vitamin D3 (400,000 IU) or Standard dose (50,000 IU), after COVID-19 diagnosis in 72 h	400, 000 IU (*n* = 127), 50,000 IU (*n* = 127)	Clear benefit in 14 days COVID-19 death (6% vs. 11%)	Early vitamin D3 (400, 000 IU) supply reduced deaths in elderly patients.
Cesur et al. (2023) (48)	Turkey	Single oral vitamin D3 (150,000 IU) or Control, after COVID vaccination	150,000 IU (*n* = 16: 14 Pfizer-BioNTech, 2 Sinovac), Control (*n* = 17: 14 Pfizer-BioNTech, 3 Sinovac)	Significant increase of serum IgG, difference between IgG and serum 25-OH-D3in supplementation period	Vitamin D3 (150,000 IU) upregulate immune response and effective in vaccine-induced antibody levels.
Sarhan et al. (2022) (49)	Egypt	Intramuscular high dose vitamin D3 (200,000 IU/d) or Oral low dose alphacalcidol (active form of vitamin D3) (40 IU/d), at least consecutive 5 days	200,000 IU/d vitD3 (*n* = 58), 40 IU/d alphacalcidol (*n* = 58)	Significantly shortened hospitalization (8.6 vs. 6.8d), reduced necessity of high-oxygen and non-invasive mechanical ventilator (67 vs. 33%), clinical improvement (45 vs. 55%), onset of sepsis (64 vs. 33%)	Vitamin D3 (200,000 IU/d) is effective in cytokine storms and fewer adverse outcomes.
Karonova et al. (2022) (50)	Russia, USA	Oral high dose vitamin D3 (50,000 IU/w), 2 weeks and (5,000 IU/d), 3 months, or Standard dose (2,000 IU/d), 3 months	50,000 IU/w + 5,000 IU/d (*n* = 45), 2,000 IU/d (*n* = 46)	Only 26% in high dose onset asymptomatic COVID-19 but twice in standard dose.	Vitamin D3 (50,000 IU/w + 5,000 IU/d) is effective and safe to achieve enough blood 25-OH-D3 level.
Karonova et al. (2022) (143)	Russia	Oral vitamin D3 (50,000 IU/d) or Control, clinical features and inflammation markers in COVID-19 patients, 1 and 8 days of hospitalization	50,000 IU/d (*n* = 56), Control (*n* = 54)	Significant difference in serum 25-OH-D3 levels (*p* < 0.001), high neutrophil and lymphocyte counts (*p* = 0.04; *p* = 0.02), low CRP level (*p* = 0.02)	Vitamin D3 (50,000 IU) increases serum 25-OH-D3 levels with positive effects.
Bychinin et al. (2022) (51)	Russia	Oral vitamin D3 (60,000 IU/w) and (5,000 IU/d), or Control, 7 weeks	60,000 IU/w + 5,000 IU/d (*n* = 55), Control (*n* = 55)	Significantly higher NK and NKT cell counts and neutrophil-to-lymphocyte ratio (NLR) on day 7	Vitamin D3 (60,000 IU/w + 5,000 IU/d) significantly increased lymphocyte numbers.
De Niet et al. (2022) (144)	Belgium	Oral vitamin D3 or Control, (25,000 IU/d), 4 days and (25,000 IU/w), maximum 6 days	25,000 IU (*n* = 50), Control (*n* = 50)	Low hospitalized rate after 7 days (19 vs. 54%; *p* = 0.0161), hospitalized patients’ numbers at day 21 (0 vs. 14), reduced oxygen supply (4 days vs. 7), significant reduction of WHO scale	Vitamin D3 (25,000 IU) improved clinical outcome in hospitalized patients.
Torres et al. (2022) (52)	Spain	Oral vitamin D3 high dose (10,000 IU/d) or Moderate dose (2,000 IU/d), 2 weeks	10,000 IU/d (*n* = 41), 2,000 IU/d (*n* = 44)	Increase of average serum 25-OH-D3 levels (29 vs.19 ng/mL; *p* < 0.0001)	Addition of vitamin D3 (10,000 IU/d) to the standard treatment shorten the period of hospitalization and improve the prognosis.
Sabico et al. (2021) (53)	Saudi Arabia	Oral vitamin D3 high dose (5,000 IU/d) or Standard dose (1,000 IU/d), middle to moderate COVID-19 patients, 2 weeks	5,000 IU/d (*n* = 36), 1,000 IU/d (*n* = 33)	Significantly increases serum 25-OH-D3 levels (*p* = 0.003)	2 weeks of daily oral vitamin D3 (5,000 IU/d) shorten the recovery time of coughing and taste loss.
van Helmond et al. (2022) (145)	USA	Oral vitamin D3 (5,000 IU/d) or Control, in healthcare workers with influenza-like illness (ILI), at least 2 months	5,000 IU/d (*n* = 255: 47 ± 12 years old, Female 99), Control (*n* = 2,827)	Significantly reduces ILI risks and non-COVID ILI incidence	Vitamin D3 (5,000 IU/d) alleviates influenza-like illness in healthcare workers.
LaRiccia et al. (2023) (54)	USA	Oral vitamin D3 (5,000 IU/d) or Control, in 9 months	5,000 IU/d (*n* = 196), Control (*n* = 1958)	Reduced healthcare utilization due to COVID-19 (rate difference: −8.47 × 10^−3^ per 1,000 person-days)	Vitamin D3 (5,000 IU/d) reduced hospitalizations due to COVID-19.
Villasis-Keever et al. (2022) (142)	Mexico	Oral vitamin D3 (4,000 IU/d) or Control, 30 days follow up	4,000 IU/d (*n* = 94), Control (*n* = 98)	Reduction of SARS-CoV-2 infection (6.4 vs. 24.5%; *p* < 0.001), lowered inflation risks, kept high serum 25-OH-D3 levels irreverent to vitD3 deficiency.	Vitamin D3 (4,000 IU) prevents SARS-CoV-2 infection.
Caballero-García et al. (2021) (139)	Spain	Oral vitamin D3 (2,000 IU /d) or Control, 6 weeks	2,000 IU/d (*n* = 15), Control (*n* = 15), Male	Optimized serum creatine kinase levels and protective effects for muscle catabolism	Vitamin D3 (2,000 IU) reduces the muscle damage indicators and improve the health status and QOL in recovery period.
Elamir et al. (2022) (146)	Israel	Oral calcitriol (active form of vitamin D3) (20 IU/d) or Control, 2 weeks	20 IU/d calcitriol (*n* = 50), Control (*n* = 50)	Increase of peripheral arterial oxygen saturation to the inspired fraction of oxygen (SaO_2_/FIO_2_ ratio) in intervention (+91.04 vs. +13.21)	Calcitriol (20 IU/d) intervention improves blood oxygen saturation in hospitalized patients.
Dilokpattanamongkol et al. (2024) (147)	Thailand	Oral alfacalcidol (active form of vitamin D3) (80 IU/d) or Control, COVID-19 patients, until discharge	80 IU/d alphacalcidol (*n* = 147), Control (*n* = 147)	Significant reduction of pneumonia severity index (*p* = 0.007) and CRP in patients over 30 mg/L (*p* < 0.001)	Addition of active vitamin D3 (80 IU/d) to the standard treatment is beneficial to the patients requiring oxygen supplementation, high dose corticosteroid therapy or patients with high CPR (> 30 mg/L).
Entrenas Castillo et al. (2020) (55)	Spain	Oral 25-OH-D3 or Control, day1 (20,000 IU), day 3 and 7 (10,000 IU), COVID hospitalized patients	20,000 IU 25-OH-D3 (*n* = 50), Control (*n* = 26)	Intervention: none died, all discharged without complications. Control: all not admitted to the ICU discharged. Of the 13 patients admitted to the ICU, two died and remaining 11 discharged.	25-OH-D3 (20,000 IU) intervention reduces the severity.
Bishop et al. (2023) (132)	USA	Oral extended-release 25-OH-D3 or Control, in COVID-19 patients, (12,000 IU/d) day 1–3 and (2,400 IU/d) day 4–27	12,000 IU 25-OH-D3 (*n* = 65), Control (*n* = 69)	Serum 25-OH-D3 > 50 ng/mL (81 vs. 15%; *p* < 0.0001)	Serum 25-OH-D3 levels became >50 ng/mL in outpatients, improve the prognosis and reduce the risk of pneumonia.
Maghbooli et al. (2021) (148)	Iran	Oral 25-OH-D3 (around 3,000–6,000 IU/d) or Control, hospitalized COVID-19 patients of blood 25-OH-D3 lower than 30 ng/mL	25-OH-D3: Control, Assigned (53:53), First month (34:24), 2nd month (24:19)	Increased lymphocyte populations and reduces neutrophil/lymphocyte ratio, low neutrophil/lymphocyte ratio is associated with ICU admission days and mortality.	Oral 25-OH-D3 upregulate immune responses through lymphocyte population and correct vitamin D deficiency in patients.
Mahjoub et al. (2024) (136)	Tunisia	Supplement (zinc, multivitamin and melatonin) or Control, treatment of COVID-19 and similar symptoms in 30 days	Intervention (*n* = 88), Control (*n* = 87)	Complete recovery (80.5 vs. 67.1%; *p* = 0.038)	Melatonin, zinc, and vitamins shorten the recovery time in and other diseases.
Reino-Gelardo et al. (2023) (149)	Spain	Food supplement (probiotics, prebiotics, vitamin D, zinc, and selenium), in hospitalized COVID-19 patients or control	Intervention (*n* = 70), Control (*n* = 69)	Shorter digestive symptoms (2.6 vs. 4.3 days; *p* = 0.001), shorter hospital stay of non-severe disease on chest X-ray patients (8.1 vs. 11.6 days; *p* = 0.007).	Food supplement (Gasteel Plus^®^) was protective factor and shorten the recovery of GI symptoms.

However, it is important to note that long-term intake of higher-than-necessary doses of vitamin D, especially with calcium, should be avoided as it can cause vitamin D toxicity. Therefore, vitamin D should be considered an immune-enhancing nutrient rather than a therapeutic agent.

**Figure 4:**
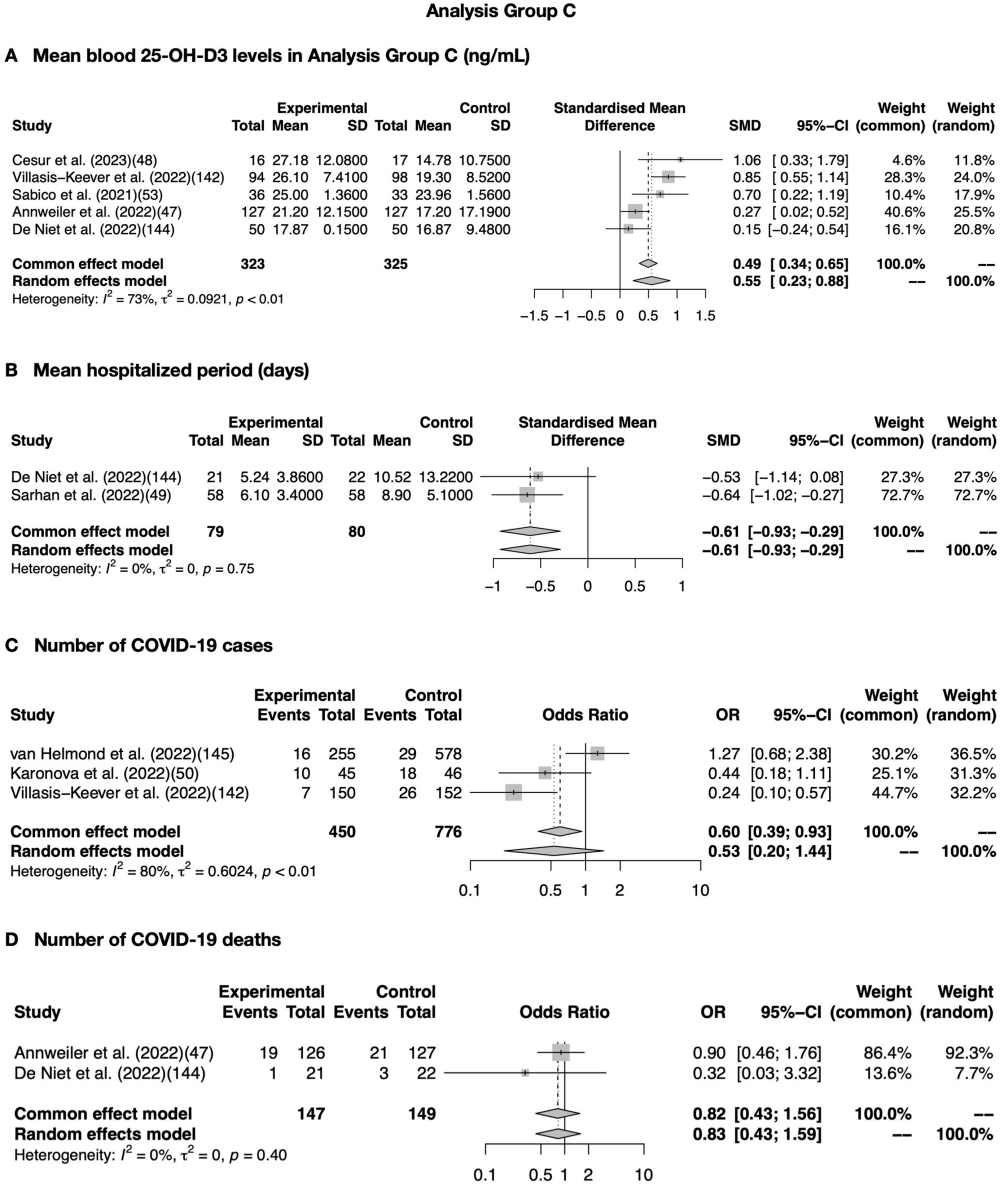
Meta-analysis of association between COVID-19 and vitamin D supplementation in Analysis Group B. Meta-analysis conducted using data extracted from articles in Table 8. **(A)** Show a meta-analysis of serum 25-OH-D levels combination of <*I*^2^ = 75%. **(B)** Shows a meta-analysis of the difference of mean hospitalized period extracted from articles shown in Table 8. **(C)** Shows a meta-analysis of COVID cases; data was shown in odds ratio. **(D)** Shows a meta-analysis of COVID deaths. *p* < 0.01 was considered as a significant difference.

In addition, a meta-analysis was performed on the 20 articles shown in Table 8, and the quantitative analysis is summarized in Figure 4. Blood 25-OH-D3 levels were found in 12 articles supplemented with vitamin D3 (10 articles) and 25-OH-D3 (two articles). Five articles showing only medians were converted into mean ± SD. The values of the five articles indicating adequate heterogeneity (*I^2^* = 73%) are shown in a forest blot (Figure 4A). Similarly, analysis of two articles showing the length of hospitalization period described in mean ± SD and total number were shown (Figure 4B). Three articles regarding COVID-19 cases (Figure 4C) and two articles regarding COVID-19 deaths (Figure 4D) contained these events; the total numbers are also illustrated. Statistically significant differences (*p* < 0.01) were observed in blood 25-OH-D3 levels (ng/mL) and number of COVID-19 cases.

#### 3.4.3 Verification of the association between COVID-19 and gut microbiota

Table 9 summarizes eight relevant RCTs out of the 12 searched for COVID and gut microbiota. All the retrieved RCTs focused on the effects of prebiotics and probiotics on COVID-19. Two RCTs from China reported a probiotic, SIM01. According to the literature, the symbiotic formulation of SIM01 contains three bacterial strains: *Bifidobacterium adolescentis*, *Bifidobacterium bifidum*, and *Bifidobacterium longum*, and three prebiotic compounds. The first study reported that SIM01 improved intestinal microbiota imbalance (56), and the second reported the alleviation of symptoms in patients with acute post-acute COVID-19 syndrome (PACS) (57). In another report, an aqueous extract of *Dendrobium officinale* (DoAE) was found to reduce inflammatory gut microbiota (58). Additionally, a Mexican study reported that probiotics containing *Lactiplantibacillus plantarum* and *Pediococcus acidilactici* enhance antibody production against COVID-19 by interacting with the host immune system (59). Similarly, a study conducted in the UK found that probiotics, including *Lactobacillus acidophilus*, *Lactobacillus plantarum*, *Bifidobacterium bifidum*, and *Bifidobacterium animalis* subsp. *lactis*, reduced the symptoms of viral upper respiratory tract infections (URTI) symptoms by 27% in overweight/obese subjects (60). In Sweden, probiotics containing *Limosilactobacillus reuteri* have been reported to increase antibody production following vaccination compared to vitamin D alone (61). In a Spanish study, probiotics and prebiotics improved the cardiometabolic profile (62), and in the USA, prebiotic fibers were shown to affect the gut microbiota associated with serum serotonin production and help improve mental health during long COVID (63).

**Table 9:** Analysis Group C: Association between COVID-19 and gut microbiota (COVID and gut microbiota) RCT.

First author, year, references	Country	Treatment	Subjects	Main findings	Outcome
Wong et al. (2023) (56)	China	Probiotics (SIM01) after initial COVID-19 vaccination within a week, 3 months	Intervention (*n* = 224), Control (*n* = 229)	SIM01 improves quality of sleep (*n* = 53 vs. 22), improvement in skin condition (*n* = 18 vs. 8), better mood (*n* = 27 vs. 13)	Probiotics SIM01 recover dysbiosis in diabetic patients and elderly in pandemic.
Lau et al. (2024) (57)	China	Probiotics (SIM01: 10 billion CFU/d), in post-acute COVID-19 syndrome (PACS) patients, 1 time/d, 6 months	Intervention (*n* = 232), Control (*n* = 231)	Recovery from fatigue (OR 2·273, 95% CI 1·520–3·397, *p* = 0·0001), memory loss (1·967, 1·271–3·044, *p* = 0·0024), difficulty in concentration (2·644, 1·687–4·143, *p* < 0·0001), gastrointestinal upset (1·995, 1·304–3·051, *p* = 0·0014), general unwellness (2·360, 1·428–3·900, *p* = 0·0008)	Probiotics SIM01 reduced several PACS symptoms.
Gao et al. (2023) (58)	China	Upregulation of immune response with *Dendrobium officinale* aquatic extract (DoAE) supplementation, in healthy subjects after COVID vaccination, 9 weeks	Intervention (*n* = 39), Control (*n* = 30)	Significant increase of physical performance, sleep, mental performance, appetite, IFN-γ production, and the number of *Faecalibacterium*	DoAE upregulate immune responses, decrease inflammatory gut microbiota and dysbiosis.
Gutiérrez-Castrellón et al. (2022) (59)	Mexico	Probiotics (*Lactiplantibacillus plantarum* KABP022, KABP023, KAPB033 strain, *Pediococcus acidilactici* KABP021 strain: total 2 × 10^9^ CFU), 30 days	Intervention (*n* = 147), Control (*n* = 146)	Significantly increased SARS-Cov-2 specific IgM and IgG	Probiotics not only changed the gut microbiota in colon but also interact with host immune system.
Mullish et al. (2021) (60)	UK	Influence of probiotics in viral upper respiratory tract infections (URTI), 6 months	BMI 25–34.9 kg/m^2^, 30–65 years old (*n* = 220)	Significantly reduced URTI symptoms by 27%, especially in subjects over 45 years old and BMI 30 kg/m^2^	Probiotics prevents viral URTI especially in overweight/obese people.
Forsgård et al. (2023) (61)	Sweden	Probiotics (*Limosilactobacillus reuteri* DSM 17938: smallest 1 × 10^8^ CFU) + vitD3 (10 μg /d), control was supplied only vitD3, 2 times/d, 6 months	Participants (*n* = 159), Completion of 3 times of research visit (*n* = 132)	In intention-to-treat (ITT) analysis, COVID positive individuals (*n* = 6) have higher serum anti-spike IgG (6,09 L vs. 111 BAU/mL) and anti-receptor binding domain IgG (928 vs. 83.7 BAU/mL)	Probiotics strengthen IgA response in mRNA based COVID vaccinated patients.
Sevillano-Jiménez et al. (2022) (62)	Spain	Nutritional education program with high symbiotic foods (dairy products, fermented foods, green-yellow vegetables, high-fiber, and whole grains), 6 months	Intervention (*n* = 23), Control (*n* = 21)	Statistical differences in all anthropometric variables, 27.4% reduction in the prevalence of metabolic syndrome risk factors, decrease in cardiovascular risk at 6 months	Probiotics improves cardio metabolic profiles in hospitalized COVID-19 patients with schizophrenia spectrum disorders.
Blackett et al. (2022) (63)	USA	Prebiotics-fibers in GI symptoms and mental health symptoms after COVID-19, 6 months	(i) Faecal samples from patients with acute COVID-19, (ii) blood samples from patients with acute COVID-19	Blood serotonin synthesis associated reduced biosynthesis of L-tryptophan by the gut microbiota affects severe GI symptoms	Reduction of serotonin signaling associated gut microbiome is associated with persistent GI symptoms and mental health in long-COVID.

Thus, the gut microbiota plays a significant role in improving COVID-19 outcomes and sequelae, as evidenced by a systematic review.

### 3.5 Analysis group D: Association between healthy diet, phytochemicals, vitamin D, and gut microbiota

#### 3.5.1 Association of polyphenols or flavonoids with gut microbiota

The results of the PubMed search for polyphenols, flavonoids, and the gut microbiota are shown in Table 10. As there were 30 RCTs reporting these associations over a 5-year period, the seven main articles from the past year are listed below.

**Table 10:** Analysis group D: Association of polyphenols or flavonoids with gut microbiota (polyphenol or flavonoids and gut microbiota) RCT last 1 year.

First author, year, references	Country	Treatment	Subjects	Main findings	Outcome
Mathrani et al. (2023) (64)	New Zealand	Rutin	Rutin supplemented yogurt 500 mg/d (*n* = 24), Rutin capsule (*n* = 25), Control (*n* = 24), 12 weeks	Fasting blood glucose has inverse relationship with butyrate-producing *Roseburia inulinivorans* abundance	First examination of after meal pancreatic β-cell function with rutin.
Jamieson et al. (2024) (65)	USA	Xanthohumol (XN)	NX 24 mg/d (*n* = 16), Control (*n* = 14), 8 weeks	Re-shape of individual taxa in an enterotype-dependent manner	Reductions in microbiota-derived bile acid metabolism specific to *Prevotella* and *Ruminococcus* enterotypes were derived.
Tosi et al. (2023) (150)	Italy, UK	Cranberry (poly)phenol	Freeze dried cranberry powder (*n* = 31), Control (*n* = 29), 12 weeks	Cranberry was associated with the changes of blood polyphenol metabolites levels	Cranberry polyphenol is associated with the health improving effects.
Lackner et al. (2024) (151)	Austria	Aronia	Natural aronia juice (*n* = 20), Control (*n* = 20), Female, twice/day, 6 weeks	Intervention group was divided into tolerant (Vt) and intolerant (Vc), Vt significantly changed microbiome diversity	Aronia juice polyphenol had personally different responses for gut microbiota.
Wattanathorn et al. (2023) (66)	Thailand	Anthocyanin	Intervention (4 g) (*n* = 23), (2 g) (*n* = 23), Control (*n* = 23), 8 weeks	Cognitive function↑, Working memory↑, Eye dryness↓, *Bifidobacterium* spp.↑	Anthocyanin holding supplement (Anthaplex) increased *Bifidobacterium* spp. and improved cognitive function and symptom of dry eyes
Kamer et al. (2023) (67)	Israel, UK, USA, France, Germany	High-polyphenol green Mediterranean diet	aFMT (*n* = 41), Control (*n* = 41), 6 months	High gut microbiota diversity participants avoid recovery of body weight increase for 8–14 months (−0.58 ± 2.4 vs. 3.18 ± 3.5 kg; *p* = 0.02)	High-polyphenol green Mediterranean diet was effective in the decrease of bodyweight in autologous-fecal-microbiota-transplantation (aFMT).
Yaskolka Meir et al. (2023) (68)	Israel, USA, France, Germany	Polyphenol rich low red/processed meet green Mediterranean diet (MED)	Green-MED (*n* = 87), MED (*n* = 81), Control (*n* = 88), Green-MED include green tea (3–4 cup/d) with Wolffia green shake (500 mL) (+800 mg/d polyphenol), Both MED groups take walnuts (28 g/d) (+440 mg/d polyphenol)	MED intervention improves DNA methylation age (mAge) - 8.9 months (*p* = 0.02)	High-polyphenol intake in MED had inverse association between biological ageing.

A study from New Zealand indicated a relationship between the intake of rutin-supplemented yogurt and an increase in the number of butyrate-producing bacteria and decrease in fasting blood glucose levels (64). In an American study, xanthohumol found in hops reduced bile acid metabolism via microbiota specific to the gut forms of *Prevotella* and *Ruminococcus* (65). Functional foods containing anthocyanins increased *Bifidobacterium* and improved cognitive function and eye dryness (66). Additionally, autologous fecal transplantation was effective in weight loss among consumers of a high-polyphenol Green Mediterranean Diet (67). An interesting finding was the inverse association between the Green Mediterranean diet and biological aging in the group with increased polyphenol intake (68).

These results highlight the significant impact of polyphenols and flavonoids on the gut microbiota composition and related health outcomes. Modulation of the gut microbiota by these compounds may offer protective benefits and improve overall health, particularly in the context of dietary interventions aimed at enhancing gut health and preventing disease.

#### 3.5.2 Association between vitamin D and gut microbiota

The results of the PubMed search for vitamin D and the gut microbiota are shown in Table 11. Several recent RCTs conducted over the past 5 years have demonstrated that vitamin D supplementation significantly affects changes in the gut microbiota. One year of supplementation with vitamin D (2,000 IU/day) in patients with colorectal cancer (CRC) resulted in a significant increase in *Leuconostoc pseudomesenteroides*, *Ruminococcus* YE78, *Faecalibacterium prausnitzii*, and *Bacteroides clarus* (69). Additionally, 16 weeks of vitamin D3 supplementation in vitamin D-deficient, overweight/obese individuals led to an increase in *Lachnospira* spp., a decrease in *Blautia* spp., and an increase in *Coprococcus* spp., while decreasing *Ruminococcus* spp. in groups with high serum vitamin D levels (70). Intramuscular vitamin D3 (200,000 IU) increased *Bifidobacteriaceae* and *Christensenellaceae* and decreased *Proteobacteria* after 8 weeks (71). Other findings suggest that increased vitamin D levels during pregnancy protect against the growth of sulfate-reducing bacteria such as *Desulfovibrio*, which are associated with chronic intestinal inflammatory disorders (72). Studies on vitamin supplementation, including that of vitamin D, have also shown increased microbial alpha diversity and short-chain fatty acids (73).

**Table 11:** Analysis group D: Association between vitamin D and gut microbiota (vitamin D and gut microbiota) RCT last 5 years.

First author, year, references	Country	Treatment	Subjects	Main findings	Outcome
Bellerba et al. (2022) (69)	Italy	Vitamin D (2,000 IU/d), 1 year	Intervention (*n* = 32), Control (*n* = 28)	*Leuconostoc pseudomesenteroides*↑, *Ruminococcus* YE78↑, *Faecalibacterium prausnitzii*↑, *Bacteroides clarus*↑	Vitamin D participates in gut microbiota formation and gut microbiota is associated with the efficacy of 25-OD-D3 in colorectal cancer (CRC) patients.
Naderpoor et al. (2019) (70)	Australia	Vitamin D3(100,000 IU), and (4,000 IU/d), every day, 16 weeks	Intervention (*n* = 14), Control (*n* = 12), Vitamin D deficiency, Overweight/obese	Genus *Lachnospira*↑, genus *Blautia*↓, In high 25-OD-D3 subjects, genus *Coprococcus*↑, genus *Ruminococcus*↓	Vitamin D3 significantly affects in several fecal gut microbiota.
Lee et al. (2022) (71)	South Korea	Intramuscular vitamin D3 (200,000 IU)	Intervention (*n* = 8), Control (*n* = 10)	In recovery, Microbial alfa diversity↑, *Proteobacteria*↓, *Lachnospiraceae*↑, *Ruminococcaceae*↑, *Akkermansiaceae*↑, *Bifidobacteriaceae*↑ After 8 weeks, *Bifidobacteriaceae*↑, *Christensenellaceae* ↑, *Proteobacteria* ↓	High dose intramuscular vitamin D3 influences gut microbiota in patients with *Clostridioides difficile* infection.
Aparicio et al. (2023) (72)	USA	Vitamin D3 (4,400 IU/d) for pregnant women	(*n* = 114)	Maternal gut microbiome is not changed by vitamin D and pregnant women have high genus *Desulfovibrio* population.	Increased vitamin D level during pregnancy could be protective against the growth of sulfur-reducing bacteria such as *Desulfovibrio*.
Pham et al. (2021) (73)	Switzerland	Vitamin A, B2, C, D, E	Vitamin A, B2, C, B2 + C, D3, E (*n* = 12) each, Control (*n* = 24)	Microbial alfa diversity↑, Fecal short fatty acid↑, Vitamin C had the largest effect	Follow-up studies with vitamins to the colon may help clarify the clinical significance of gut microbiota.

These findings highlight the significant role of vitamin D in modulating the gut microbiota, which may have implications for overall health and management of diseases related to gut health. The beneficial effects of vitamin D on the gut microbiota composition suggest its potential therapeutic application, particularly in conditions involving gut dysbiosis and inflammatory disorders.

#### 3.5.3 Relevance of phytochemicals and vitamin D

The association between vitamin D, an essential nutrient, and polyphenols and flavonoids, the main components of the phytochemical group, shown in Table 12 after a PubMed search. Several studies have highlighted synergistic effects of these nutrients.

**Table 12:** Analysis group D: Relevance of phytochemicals and vitamin D (polyphenol or flavonoids and vitamin D) RCT last 5 years.

First author, year, references	Country	Treatment	Subjects	Main findings	Outcome
Arabnezhad et al. (2022) (74)	Iran	Curcumin	Intervention (*n* = 38), Control (*n* = 38), (Curcuminoid 500 mg + piperine 5 mg), every day, from approximately 7 days before until 3 days after menstruation for three consecutive menstrual cycles	Blood 25-OH-D3↑, Aspartate aminotransferase↓, Bilirubin↓	Curcumin improved serum 25-OH-D3 levels and liver function enzyme test results in premenstrual syndrome (PMS) and dysmenorrhea women
Wong et al. (2020) (75)	Australia	Resveratrol and vitamin D3	Intervention (*n* = 73), Control (*n* = 73), twice/day, 24 months, crossover trial	Bone density in lumbar spine and neck of femur↑, Bone absorption marker: C-terminal telopeptide type-1 collagen levels↓	Bone protective effects of resveratrol were larger in subjects taking vitamin D and calcium.
Gualtieri et al. (2019) (76)	Italy	Mixed apple and bergamot (MAB) juice addition to Mediterranean diet	(*n* = 24: in 16 Female), 2 weeks	Gain in lean mass↑, Total cholesterol/HDL index↓, MIF↑, PPARγ↑, SOD1↑, VDR↑	MAB juice addition to Mediterranean diet reduced the risk of chronic non-communicative diseases (CNCDs), and increased VDR gene expression.
Federico et al. (2019) (77)	Italy	Silymarin and vitamin D	Intervention (*n* = 60), Control (*n* = 30), 6 months	Metabolic markers↓, Endothelial dysfunction↓, Oxidative stress parameters↓, worsening of disease↓, after 6 months	Silymarin and vitamin D containing supplements (RealSIL 100D^®^) improves NAFLD.
Scaturro et al. (2023) (78)	Italy	Resveratrol and vitamin D3	Combo: Rehabilitation + Supplementation (Alpha Lipoic Acid、600 mg, Acetyl-L-Carnitine 1,000 mg, Resveratrol 50 mg, Vitamin D3 800 IU), Rehabilitation alone, Supplement alone	Pain↓, QOL↑, in combo group	Combined administration of resveratrol and vitamin D3 with rehabilitation are effective in sciatica.
Marseglia et al. (2019) (79)	Italy	Quercetin, vitamin D3, *Perilla frutescens* dried seed extract holding food supplement	Intervention (*n* = 64), Control (*n* = 64), Children, 4–12 weeks of Phase II	Halved allergic rhino conjunctivitis (AR) risks (HR = 0.54)	Quercetin, vitamin D3, *Perilla frutescens* dried seed extract containing food supplement (Lertal^®^) improves childhood AR.

First, curcumin supplementation led to significant improvements in blood vitamin D levels and liver function enzyme levels in women with premenstrual syndrome (PMS) and dysmenorrhea (74). Second, the osteoprotective effect of resveratrol was greater in the participants who were supplemented with vitamin D and calcium (75). Additionally, supplementation of the Mediterranean diet with apple and bergamot juices in an Italian study reduced the risk of chronic noncommunicable diseases (CNCD) and increased VDR gene expression (76). Silymarin combined with vitamin D improves nonalcoholic fatty liver disease (NAFLD) (77). Furthermore, a combination of alpha-lipoic acid, acetyl-L-carnitine, resveratrol, and vitamin D3 supplementation with rehabilitation was effective for sciatica (78). *Perilla frutescens* dried seed extract, containing quercetin and vitamin D3, has also been shown to be effective against pediatric allergic rhinitis (79).

As described above, a link was observed between dietary factors, phytochemicals, vitamin D, and the gut microbiota, as well as between phytochemicals and vitamin D. These findings indicated that a healthy dietary pattern is an important long-term protective factor against pneumonia, including COVID-19.

## 4 Discussion

This systematic review first examined whether a healthy diet was effective against COVID-19. Japan has the longest longevity in the world, and in recent years, there has been growing awareness of the need to reduce medical costs and prevent aging, particularly by detecting and curing non-disease conditions (ME-BYO) (80). Consequently, considerable research has been conducted on healthy longevity and diet (81). Unlike the typical high-fat, high-sugar Western diet, the Japanese diet, which is similar to the Mediterranean diet, is well-known worldwide as a healthy diet, making those who consume it less prone to metabolic-related diseases and obesity (82).

In developed countries, high infection and mortality rates owing to COVID-19 have been observed, particularly among the elderly and those with underlying diseases, who constitute a large proportion of the population. Consequently, these groups are prioritized for vaccination (83). A healthy Japanese diet may be associated with lower rates of COVID-19 due to the lower prevalence of underlying lifestyle-related diseases in the population.

Africa, on the other hand, is a region with a poor food situation, including hunger and water shortages, and the economic impact of COVID lockdowns and other problems was a major concern (84). However, contrary to our expectations, the reported number of deaths due to COVID-19 did not increase significantly. One reason is that since Africa’s situation cannot be compared with that of other regions owing to underdeveloped health systems, the COVID response in Africa has been underreported. In addition, regional differences exist between the urban and non-urban areas in Africa. However, the proportion of younger people is the highest in the world, and the proportion of patients with metabolic syndrome, lifestyle-related diseases, and diseases caused by overeating is lower than in developed and emerging countries (85). This is associated with the fact that COVID-19 mortality rates were low in Africa.

Vegetables and fruits have received attention in recent years because of their high phytochemical content (86). They are referred to as the seventh nutrient, following the three macronutrients—carbohydrates, proteins, and fats—the fourth and fifth nutrients—vitamins and minerals, and the sixth nutrient–dietary fiber. Phytochemicals have gained particular attention owing to their anti-inflammatory effects and well-known antioxidant properties. Numerous studies have investigated the antibacterial, anti-viral, and anti-cancer properties of these compounds. Grape phytochemicals such as resveratrol are among the most widely studied and used compounds (87). Many other plant-derived ingredients are extensively utilized not only in foods, but also in Kampo (traditional Japanese) and other herbal medications, from aspirin to the antimalarial drug artemisinin.

Next, we examined whether blood vitamin D levels were associated with COVID-19 mortality. Recently, there have been an increasing number of reports on the immune-boosting properties of vitamin D (88). Vitamin D was named after Elmer McCollum in 1922 as the fourth vitamin, with Vindaus et al. contributing to early research. It is well-known for its role as a bone hormone and its involvement in calcium absorption in the intestinal tract. Vitamin D deficiency is well known to be associated with rickets in children (89) and osteoporosis and osteomalacia in the elderly (90).

Vitamin D toxicity can lead to hypercalcemia and calcium accumulation in blood vessels caused by excessive vitamin D intake combined with calcium intake. This effect could be reversed by preventing excessive vitamin D intake. There are two types of vitamin D: plant-derived vitamin D2, produced in mushrooms, and animal-derived vitamin D3. Vitamin D3 is produced in the body from cholesterol precursors in the skin, but the activated form, 1α,25-(OH)2-D3, has a short half-life of a few hours and is not excessive in its natural state.

It is also well known that African Americans living in temperate regions are often deficient in vitamin D3, as its production in skin cells is inhibited by high melanin levels (91). For similar reasons, vitamin D supplementation is recommended, particularly in the UK and Scandinavian countries because of the high prevalence of vitamin D deficiency at higher latitudes (92). As mentioned previously, vitamin D deficiency is much less common in mainland Africa than in other countries. A study comparing East Africa and Finland found that East Africans had a higher vitamin D intake (93), with differences in diet and sunlight exposure across regions being associated (94).

It has also been suggested that in Africa, unlike in developed countries where the population is concentrated in urban areas, there are far more opportunities for exposure to direct sunlight owing to differences in living conditions. Therefore, sufficient vitamin D is synthesized despite the high melanin pigmentation in the skin (95). This phenomenon is attributed to the fact that people living at higher latitudes lose the need for pigments that protect their bodies from direct sunlight.

In recent years, it has been noted that vitamin D deficiency is associated with compromised immunity, since vitamin D receptors (VDRs) expressed in many cells, including immune cells (96). Active vitamin D, bound to the nuclear VDR, binds to the vitamin D response elements of genes and regulates their expression of various genes. This action is particularly prominent in proinflammatory cytokine genes such as TNF-α and IL-1β, thereby providing vitamin D with anti-inflammatory properties and making it deeply involved in immune regulation (97).

There were remarkable numbers of meta-analysis in PubMed search (65 results) associated with the keywords COVID-19 and vitamin D (98). Therefore, a meta-analysis was conducted from the references in Tables 3–5, 8, and the statistical analysis of blood 25-OH-D3 levels, hospitalization period, COVID-19 cases, and deaths in relationship to COVID-19 and vitamin D. Statistically significant differences (*p* < 0.01) in blood 25-OH-D3 levels and number of COVID-19 cases were observed in this study, similar to other meta-analyses. However, it is likely that vitamin D works as supplementary regimen for daily upregulation of immune responses to avoid infections rather than the treatment of severe COVID-19.

Contrary to prior predictions, there was no significant increase in the number of deaths from COVID-19 in Africa despite the high prevalence of other infectious diseases such as AIDS and malaria (99). Although vitamin D deficiency is common in Africa, it is less prevalent than that in other regions of the world. Therefore, it is highly likely that higher average blood vitamin D levels in Africa are associated with improved survival rates. In contrast, mortality from COVID-19 was associated with blood vitamin D levels, similar to trends observed in other regions.

Finally, we examined whether COVID-19 is associated with the gut microbiota. In recent years, gut microbiota have been found to be associated with various diseases (100). The gut microbiota of the Japanese people can be categorized into five types. The gut microbiota phenotype of healthy Japanese individuals is referred to as the rural type and is characterized by high levels of *Prevotella*, which is associated with a reduced risk of various diseases (101). A study of African children found that, compared to their European counterparts, children in rural African villages had an enrichment of *Bacteroides* and a reduction of *Firmicutes*, resulting in a more diverse and healthier gut microbiota (102). This was attributed to the primitive, fiber-rich diet of Africans with healthy low *Firmicutes*/*Bacteroides* (F/B) ratio and linked to the low COVID-19 infection rates and deaths in Africa, presenting a remarkably interesting finding.

Patients with COVID-19 show reduced diversity of microbiota in the lungs, including a reduction in *Bacteroides* (103). Focusing on the gut microbiota, it was found that short-chain fatty acid-producing bacteria, mainly from the class *Clostridia* decreased, whereas opportunistic pathogens increased, resulting in leaky gut syndrome (104). Short-chain fatty acids, such as acetic acid, propionic acid, and butyric acid, are crucial for the activation of regulatory T cells (Tregs) and upregulate immunity. Furthermore, an increase in opportunistic pathogens, including mycoplasmas, has been observed in the respiratory tracts of COVID-19 patients (105). Opportunistic pathogens are normally present in the body but become pathogenic when the immune system is weakened. Thus, a link between COVID-19 and the gut microbiota has been suggested.

In addition, vitamin D helps maintain healthy gut microbiota (106). The composition of the gut microbiota varies greatly depending on the diet and can be broadly classified into obese and lean types. Obese individuals contain more *Firmicutes*, whereas lean individuals often have more *Bacteroides* (107). A typical Western diet, which is high in fat, sugar, and red meat, increases the number of obese *Firmicutes*. In contrast, a high-fiber diet rich in vegetables and fruits, such as the Japanese diet, the Mediterranean diet, the Five-a-Day diet in the USA, and vegetarian and vegan diets, increases *Bacteroidetes* (108). The Mediterranean diet, a representative healthy diet, is characterized by low intake of sweets and red meat, daily consumption of whole grains with a low glycemic index (GI), extra virgin olive oil, and approximately one glass of red wine per day. Additionally, daily physical activity is recommended as part of the Mediterranean diet pyramid.

The Japanese diet is also characterized by a high intake of foods that maintain a healthy gut microbiota, including low fat intake, high fish protein, and fermented foods (109). On the other hand, it is interesting to note that African villages have a primitive diet very high in dietary fiber, which maintains the diversity of the intestinal microbiota and a low *Firmicutes*/*Bacteroidetes* (F/B) ratio, which is considered healthy (110).

**Figure 5:**
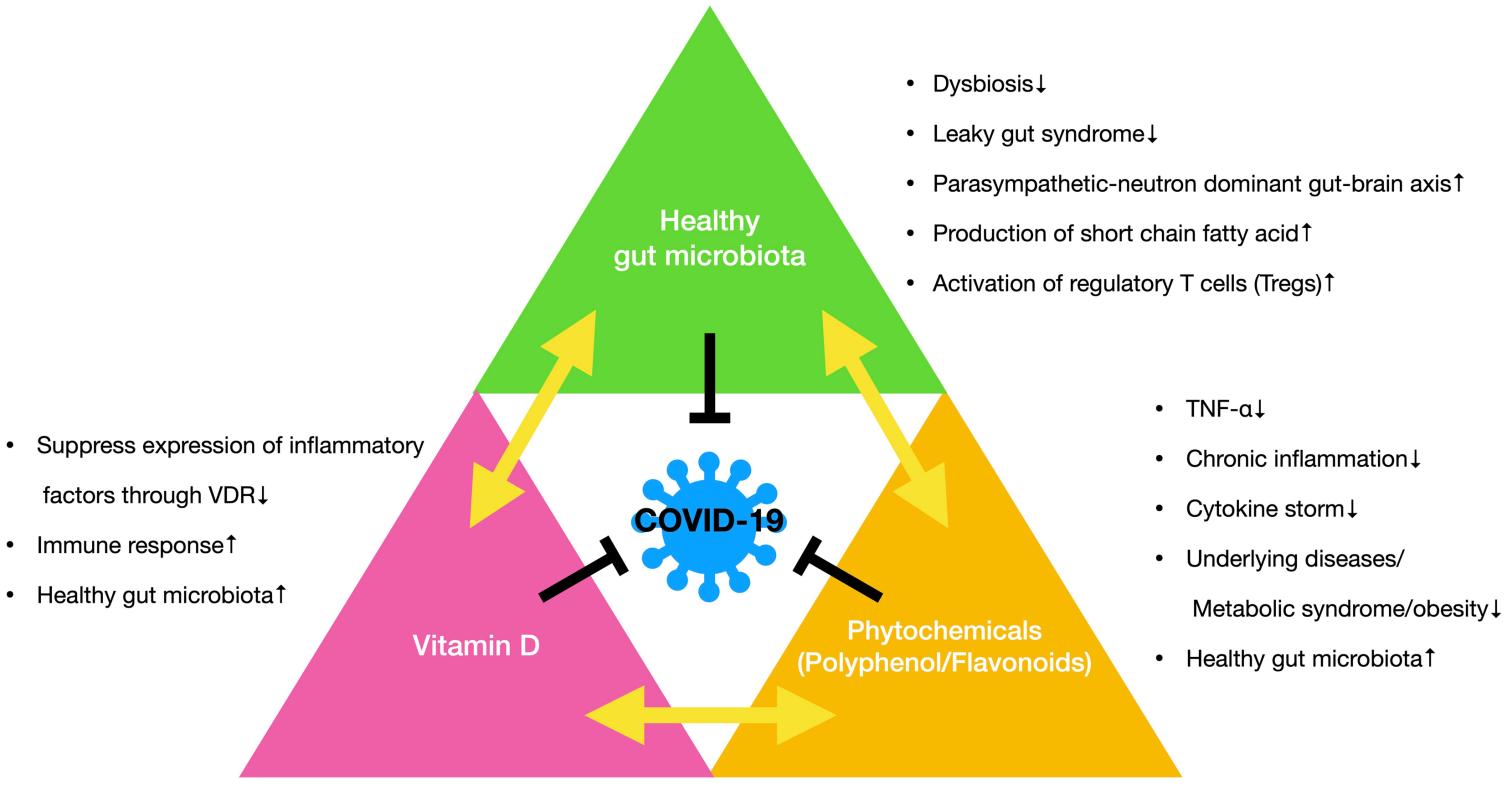
The mechanisms behind the improvement of COVID-19 responses by phytochemicals (polyphenol and flavonoids), vitamin D and gut microbiota. Phytochemicals prevent the production of proinflammatory cytokine TNF-α production; reduce chronic inflammation; prevent cytokine storms; and underlying diseases, metabolic syndrome, and obesity. Vitamin D suppresses the expression of inflammatory factors through vitamin D receptors (VDRs); upregulates immune responses; and maintains of healthy gut microbiota. Healthy gut microbiota is nurtured by both phytochemicals and vitamin D. Maintenance of healthy gut microbiota prevents dysbiosis; leaky gut syndrome; parasympathetic-neuron dominant gut-brain axis; production of short fatty acids; and upregulation of regulatory T cells (Tregs). These are associated with the prevention of the onset of COVID-19.

Figure 5 summarizes the mechanisms underlying the improvement in COVID-19 responses by phytochemicals (polyphenols and flavonoids), vitamin D, and gut microbiota. Phytochemical effects against COVID-19 via various mechanisms. First, phytochemicals not only prevent the production of proinflammatory cytokine TNF-α production but also prevent cytokine storms caused by COVID-19. The suppression of chronic inflammation prevents obesity and metabolic syndrome-related diseases, which are responsible for the onset of underlying diseases. The anti-viral effects of phytochemicals are well known. Furthermore, phytochemicals function as prebiotics and maintain a healthy gut microbiota.

Recent findings on vitamin D have demonstrated its effects on the upregulation of the immune system. Since most immune cells express vitamin D receptors (VDRs), vitamin D suppresses the transcription pathways of inflammation-associated genes and cytokine storms observed in COVID-19. Vitamin D deficiency has been observed in many deadly diseases, and their supplementation boosts immune responses. Furthermore, vitamin D intake is associated with the maintenance of healthy gut microbiota.

Maintenance of a healthy gut microbiota is associated with systemic health conditions. The onset of COVID-19 is associated with dysbiosis. This induces leaky gut syndrome, which enables the penetration of bacteria and their toxins into the bloodstream and circulation around the body, thereby inducing inflammation. Malfunction of the intestine is one of the chief symptoms of COVID-19 that worsens the condition of patients. Furthermore, the gut microbiota is associated with the maintenance of the gut-brain axis and induces a parasympathetic-neuron-dominant state related to stress reduction. In addition, short fatty acids produced by the gut microbiota activate regulatory T cells (Tregs) and prevent the manifestation of symptoms even after infection with SARS-CoV-2.

Acute pneumonia due to COVID-19 resembles sepsis caused by various infections and viruses (111). In COVID-19, SARS-CoV-2 infection causes inflammation, primarily in the lower respiratory tract, and disseminated intravascular coagulation (DIC) occurs when a cytokine storm spreads throughout the body, leading to severe symptoms and death. The mechanism is an inflammatory response, such as septic shock, triggered by infections and not just viruses. Long-term COVID-19 continues to pose a problem (112). This condition is caused by an inflammatory response that affects various parts of the body, including nerve cells, resulting in an increase in the number of aging cells. The challenge in treating long COVID, as to sepsis, is the removal of senescent cells. The antimicrobial peptide LL-37 (113), which activates innate immunity, and K-FGF (114, 115), a functional food containing phytochemicals produced from Japanese grapes (fermented grape food from Koshu), are effective in this regard.

A limitation of this study is that the medical systems in developed countries such as Japan and Africa are very different, making it difficult to determine how well recorded figures capture the actual situation. Japan has also experienced a collapse in medical systems owing to COVID-19, such as a shortage of ambulances in Tokyo; however, the medical system has been well developed. By contrast, in Africa, the population with access to hospitals is much more limited. Some reports have indicated the possibility of underestimating the impact of COVID in Africa (116). The most conceivable reason derived from the serosurveillance data is significant underdetection and underreporting (117). However, it is possible that these phenomena are applicable only to limited areas, including conflict zones (118). Second, Japan has a long life expectancy and a declining population, while in Africa, the population continues to grow and there are many children, creating completely different population pyramids. Furthermore, ACE2, the receptor for SARS-CoV-2 infection, is less expressed in young people (119), and Africans have many genetic polymorphisms, the frequency of which differs from that of people in other regions (120).

Regardless of these differences, it is necessary to consider the possibility that directly applying findings obtained from one region to another may be difficult. Moreover, with predicted future developments, healthy features such as high blood vitamin D levels and diverse gut microbiota in Africa may be lost.

Notably, in this study, COVID-19 deaths in Africa were unexpectedly low, accounting for only 2% of the global deaths. This low mortality rate is attributed not only to the high proportion of children in the population, but also to the relatively low number of people with underlying metabolic and obesity-related diseases, which are mainly caused by overeating. Additionally, high average blood vitamin D levels and, more notably, a low *Firmicutes*/*Bacteroidetes* (F/B) ratio and a highly diverse gut microbiota are contributing factors. These factors may explain the lower incidence of COVID-19 and less severe disease outcomes in Africa than in developed and emerging countries. These results are indeed very interesting.

The authors have already shown that a healthy diet containing nutrients such as phytochemicals and vitamin D is associated with a healthy gut microbiota. In this context, the present study, based on an article review, shows that phytochemicals and vitamin D are involved in the improvement of COVID-19 and its sequelae by maintaining a healthy gut microbiota. Further epidemiological studies are required to confirm these findings and explore the potential of dietary interventions to mitigate the impact of COVID-19 and improve overall public health.

## 5 Conclusion

A comparison of the Japanese and African COVID-19 responses confirmed the importance of a healthy diet. Vitamin D is related to vitamins, and its deficiency threatens the health of the body. However, it is now recognized as an immune-related hormone. Phytochemicals have also become attractive as the seventh most important nutritional source for a healthy diet in recent years. Maintaining adequate blood vitamin D levels and taking phytochemicals are associated with maintaining a healthy and diverse gut microbiota and upregulation of immune responses, which are correlated with a low mortality rate from COVID-19. This study suggests that healthy dietary patterns and nutrients are important long-term protective factors against lung diseases, including COVID-19, and may also help prevent other diseases such as sepsis caused by infections. Promoting a diet rich in phytochemicals and ensuring sufficient vitamin D intake could serve as effective strategies to enhance public health and mitigate the global impact of infectious diseases.

## Data Availability

The datasets presented in this study can be found in online repositories. The names of the repository/repositories and accession number(s) can be found at: https://center6.umin.ac.jp/cgi-open-bin/ctr_e/ctr_view.cgi?recptno=R000062073.

## Footnotes

^1^https://center6.umin.ac.jp/cgi-open-bin/ctr_e/ctr_view.cgi?recptno=R000062073

^2^https://cran.r-project.org/

## References

[1] Sayama Y Okamoto M Saito M Tamaki R Saito-Obata M Quicho RFN. Lack of zoonotic coronavirus species detected among children hospitalized with pneumonia in the Philippines. Clin Infect Dis. (2023) 77:1612–3. doi: 10.1093/cid/ciad430, PMID: 37470404 PMC10686940

[2] Halfmann PJ Hatta M Chiba S Maemura T Fan S Takeda M. Transmission of SARS-CoV-2 in domestic cats. N Engl J Med. (2020) 383:592–4. doi: 10.1056/NEJMc2013400 32402157 PMC9678187

[3] Lacy A Khan MM Deb NN Das P Igoe M Lenhart S. Geographic disparities and predictors of COVID-19 vaccination in Missouri: a retrospective ecological study. Front Public Health. (2024) 12:1329382. doi: 10.3389/fpubh.2024.1329382, PMID: 38528866 PMC10961407

[4] COVID-19 Epidemiological Update. (2024) Available at: https://www.who.int/publications/m/item/covid-19-epidemiological-update-edition-168 (Accessed June 1, 2024).

[5] WORLDOMETER. Population. (2024) Available at: https://www.worldometers.info/population/ (Accessed July 23, 2024).

[6] Nogrady B. How kids' immune systems can evade COVID. Nature. (2020) 588:382. doi: 10.1038/d41586-020-03496-7 33303982

[7] Weisberg SP Connors TJ Zhu Y Baldwin MR Lin WH Wontakal S. Distinct antibody responses to SARS-CoV-2 in children and adults across the COVID-19 clinical spectrum. Nat Immunol. (2021) 22:25–31. doi: 10.1038/s41590-020-00826-9 33154590 PMC8136619

[8] Watanabe M. The COVID-19 pandemic in Japan. Surg Today. (2020) 50:787–93. doi: 10.1007/s00595-020-02033-3, PMID: 32462468 PMC7252416

[9] Aborode AT Ogunsola SO Adeyemo AO. A crisis within a crisis: COVID-19 and hunger in African children. Am J Trop Med Hyg. (2021) 104:30–1. doi: 10.4269/ajtmh.20-1213, PMID: 33236705 PMC7790064

[10] Greene MW Roberts AP Frugé AD. Negative association between Mediterranean diet adherence and COVID-19 cases and related deaths in Spain and 23 OECD countries: an ecological study. Front Nutr. (2021) 8:591964. doi: 10.3389/fnut.2021.591964, PMID: 33748170 PMC7973012

[11] Kushida M Sugawara S Asano M Yamamoto K Fukuda S Tsuduki T. Effects of the 1975 Japanese diet on the gut microbiota in younger adults. J Nutr Biochem. (2019) 64:121–7. doi: 10.1016/j.jnutbio.2018.10.011, PMID: 30502656

[12] Sugawara S Kushida M Iwagaki Y Asano M Yamamoto K Tomata Y. The 1975 type Japanese diet improves lipid metabolic parameters in younger adults: a randomized controlled trial. J Oleo Sci. (2018) 67:599–607. doi: 10.5650/jos.ess17259, PMID: 29710042

[13] Asano M Kushida M Yamamoto K Tomata Y Tsuji I Tsuduki T. Abdominal fat in individuals with overweight reduced by consumption of a 1975 Japanese diet: a randomized controlled trial. Obesity. (2019) 27:899–907. doi: 10.1002/oby.22448 30985996

[14] Tadayon NB Rayner DG Shokraee K Shokraie K Panahi P Rastgou P. Obesity as an independent risk factor for COVID-19 severity and mortality. Cochrane Database Syst Rev. (2023) 5:CD015201. doi: 10.1002/14651858.CD015201 37222292 PMC10207996

[15] D'Ecclesiis O Gavioli C Martinoli C Raimondi S Chiocca S Miccolo C. Vitamin D and SARS-CoV2 infection, severity and mortality: a systematic review and meta-analysis. PLoS One. (2022) 17:e0268396. doi: 10.1371/journal.pone.0268396, PMID: 35793346 PMC9258852

[16] Ilie PC Stefanescu S Smith L. The role of vitamin D in the prevention of coronavirus disease 2019 infection and mortality. Aging Clin Exp Res. (2020) 32:1195–8. doi: 10.1007/s40520-020-01570-8 32377965 PMC7202265

[17] Miyamoto H Kawakami D Hanafusa N Nakanishi T Miyasaka M Furutani Y. Determination of a serum 25-Hydroxyvitamin D reference ranges in Japanese adults using fully automated liquid chromatography-tandem mass spectrometry. J Nutr. (2023) 153:1253–64. doi: 10.1016/j.tjnut.2023.01.036, PMID: 36806449

[18] Mogire RM Mutua A Kimita W Kamau A Bejon P Pettifor JM. Prevalence of vitamin D deficiency in Africa: a systematic review and meta-analysis. Lancet Glob Health. (2020) 8:e134–42. doi: 10.1016/S2214-109X(19)30457-7 31786117 PMC7024961

[19] Page MJ McKenzie JE Bossuyt PM Boutron I Hoffmann TC Mulrow CD. The PRISMA 2020 statement: an updated guideline for reporting systematic reviews. BMJ. (2021) 372:n71. doi: 10.1136/bmj.n71 33782057 PMC8005924

[20] Jastrzębska J Skalska M Radzimiński Ł López-Sánchez GF Weiss K Hill L. Changes of 25(OH)D concentration, bone resorption markers and physical performance as an effect of Sun exposure, supplementation of vitamin D and lockdown among young soccer players during a one-year training season. Nutrients. (2022) 14:521. doi: 10.3390/nu14030521 35276883 PMC8838295

[21] Populationpyramid.net. Available at: (2024). https://www.populationpyramid.net/japan/2024/ (Accessed July 29, 2024).

[22] Yuki Y Nojima M Hosono O Tanaka H Kimura Y Satoh T. Oral MucoRice-CTB vaccine for safety and microbiota-dependent immunogenicity in humans: a phase 1 randomised trial. Lancet Microbe. (2021) 2:e429–40. doi: 10.1016/S2666-5247(20)30196-8, PMID: 35544149

[23] Ao T Kikuta J Ishii M. The effects of vitamin D on immune system and inflammatory diseases. Biomol Ther. (2021) 11:1624. doi: 10.3390/biom11111624, PMID: 34827621 PMC8615708

[24] Nagata N Takeuchi T Masuoka H Aoki R Ishikane M Iwamoto N. Human gut microbiota and its metabolites impact immune responses in COVID-19 and its complications. Gastroenterology. (2023) 164:272–88. doi: 10.1053/j.gastro.2022.09.024, PMID: 36155191 PMC9499989

[25] Xue W Honda M Hibi T. Mechanisms of gastrointestinal barrier dysfunction in COVID-19 patients. World J Gastroenterol. (2023) 29:2283–93. doi: 10.3748/wjg.v29.i15.2283, PMID: 37124884 PMC10134419

[26] Li Y Watanabe E Kawashima Y Plichta DR Wang Z Ujike M. Identification of trypsin-degrading commensals in the large intestine. Nature. (2022) 609:582–9. doi: 10.1038/s41586-022-05181-3, PMID: 36071157 PMC9477747

[27] Shin-Ya M Nakashio M Ohgitani E Suganami A Kawamoto M Ichitani M. Effects of tea, catechins and catechin derivatives on omicron subvariants of SARS-CoV-2. Sci Rep. (2023) 13:16577. doi: 10.1038/s41598-023-43563-3, PMID: 37789046 PMC10547759

[28] Losso JN Losso MN Toc M Inungu JN Finley JW. The young age and plant-based diet hypothesis for low SARS-CoV-2 infection and COVID-19 pandemic in sub-Saharan Africa. Plant Foods Hum Nutr. (2021) 76:270–80. doi: 10.1007/s11130-021-00907-6 34169470 PMC8225309

[29] Houeze EA Wang Y Zhou Q Zhang H Wang X. Comparison study of Beninese and Chinese herbal medicines in treating COVID-19. J Ethnopharmacol. (2023) 308:116172. doi: 10.1016/j.jep.2023.116172 36773790 PMC9911150

[30] van Brummelen R van Brummelen AC. The potential role of resveratrol as supportive antiviral in treating conditions such as COVID-19- a formulator's perspective. Biomed Pharmacother. (2022) 148:112767. doi: 10.1016/j.biopha.2022.112767, PMID: 35240527 PMC8884665

[31] Fincham L Hohlfeld A Clarke M Kredo T McCaul M. Exploring trial publication and research waste in COVID-19 randomised trials of hydroxychloroquine, corticosteroids, and vitamin D: a meta-epidemiological cohort study. BMC Med Res Methodol. (2024) 24:19. doi: 10.1186/s12874-023-02110-4, PMID: 38262938 PMC10804507

[32] Kalichuran S van Blydenstein SA Venter M Omar S. Vitamin D status and COVID-19 severity. S Afr J Infect Dis. (2022) 37:359. doi: 10.4102/sajid.v37i1.359 35546959 PMC9082083

[33] Middelkoop K Stewart J Walker N Delport C Jolliffe DA Coussens AK. Vitamin D supplementation to prevent tuberculosis infection in South African schoolchildren: multicenter phase 3 double-blind randomized placebo-controlled trial (ViDiKids). Int J Infect Dis. (2023) 134:63–70. doi: 10.1016/j.ijid.2023.05.010 37211272

[34] Faber M Malan L Kruger HS Asare H Visser M Mukwevho T. Potential of egg as complementary food to improve nutrient intake and dietary diversity. Nutrients. (2022) 14:3396. doi: 10.3390/nu14163396, PMID: 36014905 PMC9416406

[35] Ahmadi S Mehrabi Z Zare M Ghadir S Masoumi SJ. Efficacy of Nanocurcumin as an add-on treatment for patients hospitalized with COVID-19: a double-blind, randomized clinical trial. Int J Clin Pract. (2023) 2023:5734675. doi: 10.1155/2023/5734675 37547100 PMC10403319

[36] Rodríguez-Argente F Alba-Domínguez M Díaz-Martínez MP Díaz-Vergara C Díaz-Márques B Ferrero-Ortega P. Buccopharyngeal route administered high polyphenolic olive oil and COVID-19: a pilot clinical trial. Immun Inflamm Dis. (2023) 11:e1054. doi: 10.1002/iid3.1054 37904687 PMC10587735

[37] MR MC Schnell PM Rhoda DA. Randomized double-blind placebo-controlled proof-of-concept trial of resveratrol for outpatient treatment of mild coronavirus disease (COVID-19). Sci Rep. (2022) 12:10978. doi: 10.1038/s41598-022-13920-9, PMID: 35768453 PMC9243086

[38] de Ligt M Hesselink MKC Jorgensen J Hoebers N Blaak EE Goossens GH. Resveratrol supplementation reduces ACE2 expression in human adipose tissue. Adipocytes. (2021) 10:408–11. doi: 10.1080/21623945.2021.1965315 PMC8381800 34402717

[39] Shohan M Nashibi R Mahmoudian-Sani MR Abolnezhadian F Ghafourian M Alavi SM. The therapeutic efficacy of quercetin in combination with antiviral drugs in hospitalized COVID-19 patients: a randomized controlled trial. Eur J Pharmacol. (2022) 914:174615. doi: 10.1016/j.ejphar.2021.174615 34863994 PMC8638148

[40] Aryan H Farahani RH Chamanara M Elyasi S Jaafari MR Haddad M. Evaluation of the efficacy of oral nano-silymarin formulation in hospitalized patients with COVID-19: a double-blind placebo-controlled clinical trial. Phytother Res. (2022) 36:3924–31. doi: 10.1002/ptr.7537, PMID: 35859298 PMC9349546

[41] Versace V Ortelli P Dezi S Ferrazzoli D Alibardi A Bonini I. Co-ultramicronized palmitoylethanolamide/luteolin normalizes GABA(B)-ergic activity and cortical plasticity in long COVID-19 syndrome. Clin Neurophysiol. (2023) 145:81–8. doi: 10.1016/j.clinph.2022.10.017 36455453 PMC9650483

[42] Di Stadio A Gallina S Cocuzza S De Luca P Ingrassia A Oliva S. Treatment of COVID-19 olfactory dysfunction with olfactory training, palmitoylethanolamide with luteolin, or combined therapy: a blinded controlled multicenter randomized trial. Eur Arch Otorrinolaringol. (2023) 280:4949–61. doi: 10.1007/s00405-023-08085-8, PMID: 37380908 PMC10562315

[43] De Luca P Camaioni A Marra P Salzano G Carriere G Ricciardi L. Effect of ultra-micronized Palmitoylethanolamide and Luteolin on olfaction and memory in patients with long COVID: results of a longitudinal study. Cells. (2022) 11:2552. doi: 10.3390/cells11162552, PMID: 36010630 PMC9406356

[44] Di Stadio A D'Ascanio L Vaira LA Cantone E De Luca P Cingolani C. Ultramicronized Palmitoylethanolamide and Luteolin supplement combined with olfactory training to treat post-COVID-19 olfactory impairment: a multi-center double-blinded randomized placebo-controlled clinical trial. Curr Neuropharmacol. (2022) 20:2001–12. doi: 10.2174/1570159X20666220420113513, PMID: 35450527 PMC9886808

[45] D'Ascanio L Vitelli F Cingolani C Maranzano M Brenner MJ Di Stadio A. Randomized clinical trial "olfactory dysfunction after COVID-19: olfactory rehabilitation therapy vs. intervention treatment with Palmitoylethanolamide and Luteolin": preliminary results. Eur Rev Med Pharmacol Sci. (2021) 25:4156–62. doi: 10.26355/eurrev_202106_26059, PMID: 34156697

[46] Carrouel F Valette M Gadea E Esparcieux A Illes G Langlois ME. Use of an antiviral mouthwash as a barrier measure in the SARS-CoV-2 transmission in adults with asymptomatic to mild COVID-19: a multicentre, randomized, double-blind controlled trial. Clin Microbiol Infect. (2021) 27:1494–501. doi: 10.1016/j.cmi.2021.05.028, PMID: 34044151 PMC8142805

[47] Annweiler C Beaudenon M Gautier J Gonsard J Boucher S Chapelet G. High-dose versus standard-dose vitamin D supplementation in older adults with COVID-19 (COVIT-TRIAL): a multicenter, open-label, randomized controlled superiority trial. PLoS Med. (2022) 19:e1003999. doi: 10.1371/journal.pmed.1003999, PMID: 35639792 PMC9154122

[48] Cesur F Atasever Z Özoran Y. Impact of vitamin D3 supplementation on COVID-19 vaccine response and immunoglobulin G antibodies in deficient women: a randomized controlled trial. Vaccine. (2023) 41:2860–7. doi: 10.1016/j.vaccine.2023.03.046, PMID: 37003908 PMC10040353

[49] Sarhan N Abou Warda AE Sarhan RM Boshra MS Mostafa-Hedeab G Alruwaili BF. Evidence for the efficacy of a high dose of vitamin D on the hyperinflammation state in moderate-to-severe COVID-19 patients: a randomized clinical trial. Medicina. (2022) 58:1358. 36295519 10.3390/medicina58101358 PMC9609310

[50] Karonova TL Chernikova AT Golovatyuk KA Bykova ES Grant WB Kalinina OV. Vitamin D intake may reduce SARS-CoV-2 infection morbidity in health care workers. Nutrients. (2022) 14:505. doi: 10.3390/nu14030505, PMID: 35276863 PMC8839300

[51] Bychinin MV Klypa TV Mandel IA Yusubalieva GM Baklaushev VP Kolyshkina NA. Effect of vitamin D3 supplementation on cellular immunity and inflammatory markers in COVID-19 patients admitted to the ICU. Sci Rep. (2022) 12:18604. doi: 10.1038/s41598-022-22045-y, PMID: 36329227 PMC9632570

[52] Torres M Casado G Vigón L Rodríguez-Mora S Mateos E Ramos-Martín F. Multidisciplinary group of study of COVID-19 (MGS-COVID); contributing members of the multidisciplinary group of study of COVID-19 (in alphabetical order). Changes in the immune response against SARS-CoV-2 in individuals with severe COVID-19 treated with high dose of vitamin D. Biomed Pharmacother. (2022) 150:112965. doi: 10.1016/j.biopha.2022.112965 35468580 PMC9008199

[53] Sabico S Enani MA Sheshah E Aljohani NJ Aldisi DA Alotaibi NH. Effects of a 2-week 5000 IU versus 1000 IU vitamin D3 supplementation on recovery of symptoms in patients with mild to moderate COVID-19: a randomized clinical trial. Nutrients. (2021) 13:2170. doi: 10.3390/nu13072170, PMID: 34202578 PMC8308273

[54] La Riccia PJ Cafaro T John D van Helmond N Mitrev LV Bandomer B. Healthcare costs and healthcare utilization outcomes of vitamin D3 supplementation at 5000 IU daily during a 10.9 month observation period within a pragmatic randomized clinical trial. Nutrients. (2023) 15:4435. doi: 10.3390/nu15204435 37892510 PMC10609978

[55] Entrenas CM Entrenas Costa LM Vaquero Barrios JM Alcalá Díaz JF López MJ Bouillon R. Effect of calcifediol treatment and best available therapy versus best available therapy on intensive care unit admission and mortality among patients hospitalized for COVID-19: a pilot randomized clinical study. J Steroid Biochem Mol Biol. (2020) 203:105751. doi: 10.1016/j.jsbmb.2020.105751, PMID: 32871238 PMC7456194

[56] Wong MCS Zhang L Ching JYL Mak JWY Huang J Wang S. Effects of gut microbiome modulation on reducing adverse health outcomes among elderly and diabetes patients during the COVID-19 pandemic: a randomised, double-blind, placebo-controlled trial (IMPACT study). Nutrients. (1982) 15:15. doi: 10.3390/nu15081982 PMC10143994 37111201

[57] Lau RI Su Q Lau ISF Ching JYL Wong MCS Lau LHS. A synbiotic preparation (SIM01) for post-acute COVID-19 syndrome in Hong Kong (RECOVERY): a randomised, double-blind, placebo-controlled trial. Lancet Infect Dis. (2024) 24:256–65. doi: 10.1016/S1473-3099(23)00685-0 38071990

[58] Gao X Ye T Lei Y Zhang Q Luo Y Yang H. Dendrobium officinale aqueous extract influences the immune response following vaccination against SARS-CoV-2. Biomed Pharmacother. (2023) 162:114702. doi: 10.1016/j.biopha.2023.114702, PMID: 37062221 PMC10099150

[59] Gutiérrez-Castrellón P Gandara-Martí T Abreu AT AY Nieto-Rufino CD López-Orduña E Jiménez-Escobar I. Probiotic improves symptomatic and viral clearance in COVID 19 outpatients: a randomized, quadruple-blinded, placebo-controlled trial. Gut Microbes. (2022) 14:2018899. doi: 10.1080/19490976.2021.2018899, PMID: 35014600 PMC8757475

[60] Mullish BH Marchesi JR McDonald JAK Pass DA Masetti G Michael DR. Probiotics reduce self-reported symptoms of upper respiratory tract infection in overweight and obese adults: should we be considering probiotics during viral pandemics? Gut Microbes. (2021) 13:1–9. doi: 10.1080/19490976.2021.1900997 PMC8007143 33764850

[61] Forsgård RA Rode J Lobenius-Palmér K Kamm A Patil S Tacken MGJ. Limosilactobacillus reuteri DSM 17938 supplementation and SARS-CoV-2 specific antibody response in healthy adults: a randomized, triple-blinded, placebo-controlled trial. Gut Microbes. (2023) 15:2229938. doi: 10.1080/19490976.2023.2229938 37401761 PMC10321188

[62] Sevillano-Jiménez A Romero-Saldaña M Carrascal-Laso L García-Rodríguez M Molina-Luque R Molina-Recio G. Impact of high prebiotic and probiotic dietary education in the SARS-CoV-2 era: improved cardio-metabolic profile in schizophrenia spectrum disorders. BMC Psychiatr. (2022) 22:781. doi: 10.1186/s12888-022-04426-9, PMID: 36510155 PMC9743108

[63] Blackett JW Sun Y Purpura L Margolis KG Elkind MSV O'Byrne S. Decreased gut microbiome tryptophan metabolism and serotonergic signaling in patients with persistent mental health and gastrointestinal symptoms after COVID-19. Clin Transl Gastroenterol. (2022) 13:e00524. doi: 10.14309/ctg.0000000000000524 36049050 PMC9624499

[64] Mathrani A Yip W Sequeira-Bisson IR Barnett D Stevenson O Taylor MW. Effect of a 12-week polyphenol Rutin intervention on markers of pancreatic β-cell function and gut microbiota in adults with overweight without diabetes. Nutrients. (2023) 15:3360. doi: 10.3390/nu15153360, PMID: 37571297 PMC10420824

[65] Jamieson PE Smart EB Bouranis JA Choi J Danczak RE Wong CP. Gut enterotype-dependent modulation of gut microbiota and their metabolism in response to xanthohumol supplementation in healthy adults. Gut Microbes. (2024) 16:2315633. doi: 10.1080/19490976.2024.2315633 38358253 PMC10878022

[66] Wattanathorn J Tong-Un T Thukham-Mee W Paholpak P Rangseekhajee P. A randomized, double-blind, placebo-controlled study of an anthocyanin-rich functional ingredient on cognitive function and eye dryness in late adulthood volunteers: roles of epigenetic and gut microbiome modulations. Nutrients. (2023) 15:3499. doi: 10.3390/nu15163499, PMID: 37630690 PMC10459889

[67] Kamer O Rinott E Tsaban G Kaplan A Yaskolka MA Zelicha H. Successful weight regain attenuation by autologous fecal microbiota transplantation is associated with non-core gut microbiota changes during weight loss; randomized controlled trial. Gut Microbes. (2023) 15:2264457. doi: 10.1080/19490976.2023.2264457, PMID: 37796016 PMC10557561

[68] Yaskolka MA Keller M Hoffmann A Rinott E Tsaban G Kaplan A. The effect of polyphenols on DNA methylation-assessed biological age attenuation: the DIRECT PLUS randomized controlled trial. BMC Med. (2023) 21:364. doi: 10.1186/s12916-023-03067-3, PMID: 37743489 PMC10519069

[69] Bellerba F Serrano D Johansson H Pozzi C Segata N Nabi NA. Colorectal cancer, vitamin D and microbiota: a double-blind phase II randomized trial (ColoViD) in colorectal cancer patients. Neoplasia. (2022) 34:100842. doi: 10.1016/j.neo.2022.100842, PMID: 36279751 PMC9594107

[70] Naderpoor N Mousa A Fernanda Gomez Arango L Barrett HL Dekker NM de Courten B. Effect of vitamin D supplementation on Faecal microbiota: a randomised clinical trial. Nutrients. (2019) 11:2888. doi: 10.3390/nu11122888, PMID: 31783602 PMC6950585

[71] Lee SH Park HK Kang CD Choi DH Park SC Park JM. High dose intramuscular vitamin D3 supplementation impacts the gut microbiota of patients with Clostridioides difficile infection. Front Cell Infect Microbiol. (2022) 12:904987. doi: 10.3389/fcimb.2022.904987 35774395 PMC9239168

[72] Aparicio A Gold DR Weiss ST Litonjua AA Lee-Sarwar K Liu YY. Association of vitamin D Level and maternal gut microbiome during pregnancy: findings from a randomized controlled trial of antenatal vitamin D supplementation. Nutrients. (2023) 15:2059. doi: 10.3390/nu15092059, PMID: 37432235 PMC10181263

[73] Pham VT Fehlbaum S Seifert N Richard N Bruins MJ Sybesma W. Effects of colon-targeted vitamins on the composition and metabolic activity of the human gut microbiome-a pilot study. Gut Microbes. (2021) 13:1–20. doi: 10.1080/19490976.2021.1875774 PMC7899684 33615992

[74] Arabnezhad L Mohammadifard M Rahmani L Majidi Z Ferns GA Bahrami A. Effects of curcumin supplementation on vitamin D levels in women with premenstrual syndrome and dysmenorrhea: a randomized controlled study. BMC Complement Med Ther. (2022) 22:19. doi: 10.1186/s12906-022-03515-2, PMID: 35065636 PMC8784001

[75] Wong RH Thaung Zaw JJ Xian CJ Howe PR. Regular supplementation with resveratrol improves bone mineral density in postmenopausal women: a randomized. Placebo-Controlled Trial J Bone Miner Res. (2020) 35:2121–31. doi: 10.1002/jbmr.4115, PMID: 32564438 PMC7689937

[76] Gualtieri P Marchetti M Frank G Smeriglio A Trombetta D Colica C. Antioxidant-enriched diet on oxidative stress and inflammation gene expression: a randomized controlled trial. Genes. (2023) 14:206. doi: 10.3390/genes14010206, PMID: 36672947 PMC9859217

[77] Federico A Dallio M Masarone M Di Sarno R Tuccillo C Cossiga V. Evaluation of the effect derived from Silybin with vitamin D and vitamin E administration on clinical, metabolic, endothelial dysfunction, oxidative stress parameters, and serological worsening markers in nonalcoholic fatty liver disease patients. Oxidative Med Cell Longev. (2019) 2019:8742075. doi: 10.1155/2019/8742075 PMC6815609 31737175

[78] Scaturro D Vitagliani F Tomasello S Sconza C Respizzi S Letizia MG. Combined rehabilitation with alpha lipoic acid, acetyl-L-carnitine, resveratrol, and cholecalciferolin discogenic sciatica in young people: a randomized clinical trial. Medicina. (2023) 59:2197. doi: 10.3390/medicina59122197, PMID: 38138300 PMC10744495

[79] Marseglia G Licari A Leonardi S Papale M Zicari AM Schiavi L. A polycentric, randomized, parallel-group, study on Lertal^®^, a multicomponent nutraceutical, as preventive treatment in children with allergic rhinoconjunctivitis: phase II. Ital J Pediatr. (2019) 45:84. doi: 10.1186/s13052-019-0678-y 31319883 PMC6637471

[80] Wu X Le TK Maeda-Minami A Yoshino T Horiba Y Mimura M. Relationship between conventional medicine chapters in ICD-10 and Kampo pattern diagnosis: a cross-sectional study. Front Pharmacol. (2021) 12:751403. doi: 10.3389/fphar.2021.751403, PMID: 34987389 PMC8721141

[81] Murakami K Shinozaki N Livingstone MBE Yuan X Tajima R Matsumoto M. Associations of food choice values and food literacy with overall diet quality: a nationwide cross-sectional study in Japanese adults. Br J Nutr. (2023) 130:1795–805. doi: 10.1017/S000711452300082X, PMID: 37017207 PMC10587391

[82] Santa K Kumazawa Y Watanabe K Nagaoka I. The recommendation of the Mediterranean-styled Japanese diet for healthy longevity. Endocr Metab Immune Disord Drug Targets. (2024). doi: 10.2174/0118715303280097240130072031 [Epub ahead of print]. 38343059

[83] Chilamakuri R Agarwal S. COVID-19: characteristics and therapeutics. Cells. (2021) 10:206. doi: 10.3390/cells10020206, PMID: 33494237 PMC7909801

[84] Rabbi MF Oláh J Popp J Máté D Kovács S. Food security and the COVID-19 crisis from a consumer buying behaviour perspective-the case of Bangladesh. Food Secur. (2021) 10:3073. doi: 10.3390/foods10123073 PMC8701356 34945624

[85] Adom T De Villiers A Puoane T Kengne AP. A scoping review of policies related to the prevention and control of overweight and obesity in Africa. Nutrients. (2021) 13:4028. doi: 10.3390/nu13114028, PMID: 34836281 PMC8625107

[86] Santa K Kumazawa Y Nagaoka I. The potential use of grape phytochemicals for preventing the development of intestine-related and subsequent inflammatory diseases. Endocr Metab Immune Disord Drug Targets. (2019) 19:794–802. doi: 10.2174/1871530319666190529105226, PMID: 31142251

[87] Odai T Terauchi M Kato K Hirose A Miyasaka N. Effects of grape seed proanthocyanidin extract on vascular endothelial function in participants with prehypertension: a randomized, double-blind. Placebo-Controlled Study Nutrients. (2019) 11:2844. doi: 10.3390/nu11122844 31757033 PMC6950399

[88] Santa K Kumazawa Y Watanabe K Nagaoka I. The potential use of vitamin D3 and phytochemicals for their anti-ageing effects. Int J Mol Sci. (2024) 25:2125. doi: 10.3390/ijms25042125, PMID: 38396804 PMC10889119

[89] Miller WL Imel EA. Rickets, vitamin D, and ca/P metabolism. Horm Res Paediatr. (2022) 95:579–92. doi: 10.1159/000527011, PMID: 36446330

[90] Bouillon R Marcocci C Carmeliet G Bikle D White JH Dawson-Hughes B. Skeletal and extraskeletal actions of vitamin D: current evidence and outstanding questions. Endocr Rev. (2019) 40:1109–51. doi: 10.1210/er.2018-00126, PMID: 30321335 PMC6626501

[91] Itkonen ST Andersen R Björk AK Brugård KÅ Eneroth H Erkkola M. Vitamin D status and current policies to achieve adequate vitamin D intake in the Nordic countries. Scand J Public Health. (2021) 49:616–27. doi: 10.1177/1403494819896878 31916497

[92] Cashman KD Kiely ME Andersen R Grønborg IM Madsen KH Nissen J. Individual participant data (IPD)-level meta-analysis of randomised controlled trials with vitamin D-fortified foods to estimate dietary reference values for vitamin D. Eur J Nutr. (2021) 60:939–59. doi: 10.1007/s00394-020-02298-x 32556447

[93] Adebayo FA Itkonen ST Öhman T Skaffari E Saarnio EM Erkkola M. Vitamin D intake, serum 25-hydroxyvitamin D status and response to moderate vitamin D3 supplementation: a randomised controlled trial in east African and Finnish women. Br J Nutr. (2018) 119:431–41. doi: 10.1017/S000711451700397X 29498350

[94] Carlberg C. Nutrigenomics of vitamin D. Nutrients. (2019) 11:676. doi: 10.3390/nu11030676, PMID: 30901909 PMC6470874

[95] Hanel A Carlberg C. Skin colour and vitamin D: an update. Exp Dermatol. (2020) 29:864–75. doi: 10.1111/exd.14142 32621306

[96] Lowe KE Maiyar AC Norman AW. Vitamin D-mediated gene expression. Crit Rev Eukaryot Gene Expr. (1992) 2:65–109. PMID: 1543898

[97] Santa K Watanabe K Kumazawa Y Nagaoka I. Phytochemicals and vitamin D for a healthy life and prevention of diseases. Int J Mol Sci. (2023) 24:12167. doi: 10.3390/ijms241512167, PMID: 37569540 PMC10419318

[98] Hosseini B El Abd A Ducharme FM. Effects of vitamin D supplementation on COVID-19 related outcomes: a systematic review and meta-analysis. Nutrients. (2022) 14:2134. doi: 10.3390/nu14102134 35631275 PMC9147949

[99] Bell D Schultz HK. Relative burdens of the COVID-19, malaria, tuberculosis, and HIV/AIDS epidemics in sub-Saharan Africa. Am J Trop Med Hyg. (2021) 105:1510–5. doi: 10.4269/ajtmh.21-0899, PMID: 34634773 PMC8641365

[100] Santa K. Healthy diet, grape phytochemicals, and vitamin D: preventing chronic inflammation and keeping good microbiota. Endocr Metab Immune Disord Drug Targets. (2023) 23:777–800. doi: 10.2174/1871530323666221017151705, PMID: 36263483

[101] Takagi T Inoue R Oshima A Sakazume H Ogawa K Tominaga T. Typing of the gut microbiota community in Japanese subjects. Microorganisms. (2022) 10:664. doi: 10.3390/microorganisms10030664, PMID: 35336239 PMC8954045

[102] De Filippo C Cavalieri D Di Paola M Ramazzotti M Poullet JB Massart S. Impact of diet in shaping gut microbiota revealed by a comparative study in children from Europe and rural Africa. Proc Natl Acad Sci USA. (2010) 107:14691–6. doi: 10.1073/pnas.1005963107, PMID: 20679230 PMC2930426

[103] Yamamoto S Saito M Tamura A Prawisuda D Mizutani T Yotsuyanagi H. The human microbiome and COVID-19: a systematic review. PLoS One. (2023) 16:e0253293. doi: 10.1371/journal.pone.0253293 PMC8221462 34161373

[104] Mizutani T Ishizaka A Koga M Ikeuchi K Saito M Adachi E. Correlation analysis between gut microbiota alterations and the cytokine response in patients with coronavirus disease during hospitalization. Microbiol Spectr. (2022) 10:e0168921. doi: 10.1128/spectrum.01689-21, PMID: 35254122 PMC9045125

[105] Reuben RC Beugnon R Jurburg SD. COVID-19 alters human microbiomes: a meta-analysis. Front Cell Infect Microbiol. (2023) 13:1211348. doi: 10.3389/fcimb.2023.1211348 37600938 PMC10433767

[106] Ghareghani M Reiter RJ Zibara K Farhadi N. Latitude, vitamin D, melatonin, and gut microbiota act in concert to initiate multiple sclerosis: a New mechanistic pathway. Front Immunol. (2018) 9:2484. doi: 10.3389/fimmu.2018.02484, PMID: 30459766 PMC6232868

[107] Turnbaugh PJ Ley RE Mahowald MA Magrini V Mardis ER Gordon JI. An obesity-associated gut microbiome with increased capacity for energy harvest. Nature. (2006) 444:1027–31. doi: 10.1038/nature05414 17183312

[108] Hurtado-Barroso S Trius-Soler M Lamuela-Raventós RM Zamora-Ros R. Vegetable and fruit consumption and prognosis among Cancer survivors: a systematic review and Meta-analysis of cohort studies. Adv Nutr. (2020) 11:1569–82. doi: 10.1093/advances/nmaa082, PMID: 32717747 PMC7666913

[109] Takabayashi S Okada E Hirata T Takimoto H Nakamura M Sasaki S. Nutritional adequacy assessment of the Japanese diet using the number of dishes compared to existing dietary diversity indices: a cross-sectional analysis from the 2012 national health and nutrition survey, Japan. J Nutr Sci Vitaminol. (2023) 69:197–205. doi: 10.3177/jnsv.69.197, PMID: 37394425

[110] Kumar G Bhadury P. Exploring the influences of geographical variation on sequence signatures in the human gut microbiome. J Genet. (2023) 102:51. doi: 10.1007/s12041-023-01448-4 38073168

[111] Koçak TZ Kayaaslan B Mer M. COVID-19 and Sepsis. Turk J Med Sci. (2021) 51:3301–11. doi: 10.3906/sag-2108-239, PMID: 34590796 PMC8771020

[112] Klein J Wood J Jaycox JR Dhodapkar RM Lu P Gehlhausen JR. Distinguishing features of long COVID identified through immune profiling. Nature. (2023) 623:139–48. doi: 10.1038/s41586-023-06651-y, PMID: 37748514 PMC10620090

[113] Nagaoka I Tamura H Reich J. Therapeutic potential of cathelicidin peptide LL-37, an antimicrobial agent, in a murine Sepsis model. Int J Mol Sci. (2020) 21:5973. doi: 10.3390/ijms21175973 32825174 PMC7503894

[114] Kawaguchi K Kikuchi S Hasunuma R Maruyama H Yoshikawa T Kumazawa Y. A citrus flavonoid hesperidin suppresses infection-induced endotoxin shock in mice. Biol Pharm Bull. (2004) 27:679–83. doi: 10.1248/bpb.27.679, PMID: 15133244

[115] Kumazawa Y Takimoto H Matsumoto T Kawaguchi K. Potential use of dietary natural products, especially polyphenols, for improving type-1 allergic symptoms. Curr Pharm Des. (2014) 20:857–63. doi: 10.2174/138161282006140220120344, PMID: 23701564

[116] Gill CJ Mwananyanda L Mac Leod WB Kwenda G Pieciak RC Etter L. What is the prevalence of COVID-19 detection by PCR among deceased individuals in Lusaka, Zambia? A postmortem surveillance study. BMJ Open. (2022) 12:e066763. doi: 10.1136/bmjopen-2022-066763 PMC9729848 36600354

[117] Kogan NE Gantt S Swerdlow D Viboud C Semakula M Lipsitch M. Leveraging Serosurveillance and postmortem surveillance to quantify the impact of coronavirus disease 2019 in Africa. Clin Infect Dis. (2023) 76:424–32. doi: 10.1093/cid/ciac897 36196586 PMC9619616

[118] Watson OJ Alhaffar M Mehchy Z Whittaker C Akil Z Brazeau NF. Leveraging community mortality indicators to infer COVID-19 mortality and transmission dynamics in Damascus, Syria. Nat Commun. (2021) 12:2394. doi: 10.1038/s41467-021-22474-9 33888698 PMC8062464

[119] Zimmermann P Curtis N. Why is COVID-19 less severe in children? A review of the proposed mechanisms underlying the age-related difference in severity of SARS-CoV-2 infections. Arch Dis Child. (2020) 106:429–39. doi: 10.1136/archdischild-2020-320338 33262177

[120] Zhang C Verma A Feng Y Melo MCR McQuillan M Hansen M. Impact of natural selection on global patterns of genetic variation and association with clinical phenotypes at genes involved in SARS-CoV-2 infection. Proc Natl Acad Sci USA. (2022) 119:e2123000119. doi: 10.1073/pnas.2123000119, PMID: 35580180 PMC9173769

[121] Steigmann L Maekawa S Sima C Travan S Wang CW Giannobile WV. Biosensor and lab-on-a-chip biomarker-identifying technologies for oral and periodontal diseases. Front Pharmacol. (2020) 11:588480. doi: 10.3389/fphar.2020.588480 33343358 PMC7748088

[122] Koyama S Kondo K Ueha R Kashiwadani H Heinbockel T. Possible use of phytochemicals for recovery from COVID-19-induced anosmia and Ageusia. Int J Mol Sci. (2021) 22:8912. doi: 10.3390/ijms22168912, PMID: 34445619 PMC8396277

[123] Babszky G Torma F Aczel D Bakonyi P Gombos Z Feher J. COVID-19 infection alters the microbiome: elite athletes and sedentary patients have similar bacterial Flora. Genes. (2021) 12:1577. doi: 10.3390/genes12101577, PMID: 34680972 PMC8536180

[124] Golabi S Ghasemi S Adelipour M Bagheri R Suzuki K Wong A. Oxidative stress and inflammatory status in COVID-19 outpatients: a health center-based analytical cross-sectional study. Antioxidants. (2022) 11:606. doi: 10.3390/antiox11040606 35453291 PMC9024445

[125] Nagai M Moriyama M Ishii C Mori H Watanabe H Nakahara T. High body temperature increases gut microbiota-dependent host resistance to influenza a virus and SARS-CoV-2 infection. Nat Commun. (2023) 14:3863. doi: 10.1038/s41467-023-39569-0, PMID: 37391427 PMC10313692

[126] Aina OO Okoyenta OC Okolo CA Kareem KO Ajibaye O Adeogun AO. Acute and subacute oral toxicity characterization and safety assessment of COVID organics^®^ (Madagascar's anti-COVID herbal tea) in animal models. Ann Afr Med. (2023) 22:481–8. doi: 10.4103/aam.aam_112_21, PMID: 38358149 PMC10775938

[127] Ghosh S Al-Sharify ZT Maleka MF Onyeaka H Maleke M Maolloum A. Propolis efficacy on SARS-COV viruses: a review on antimicrobial activities and molecular simulations. Environ Sci Pollut Res Int. (2022) 29:58628–47. doi: 10.1007/s11356-022-21652-6, PMID: 35794320 PMC9258455

[128] Fadaka AO Sibuyi NRS Martin DR Klein A Madiehe A Meyer M. Development of effective therapeutic molecule from natural sources against coronavirus protease. Int J Mol Sci. (2021) 22:9431. doi: 10.3390/ijms22179431 34502340 PMC8430653

[129] Zekeya N Mamiro B Ndossi H Kilonzo M Kisingo A Mtambo M. Screening and evaluation of cytotoxicity and antiviral effects of secondary metabolites from water extracts of *Bersama abyssinica* against SARS-CoV-2 Delta. BMC Complement. Med. Ther. (2022) 22:280. doi: 10.1186/s12906-022-03754-3, PMID: 36289484 PMC9598020

[130] Tiwari SK Dicks LMT Popov IV Karaseva A Ermakov AM Suvorov A. Probiotics at war against viruses: what is missing from the picture? Front Microbiol. (2020) 11:1877. doi: 10.3389/fmicb.2020.01877 32973697 PMC7468459

[131] Mariani J Antonietti L Tajer C Ferder L Inserra F Sanchez CM. High-dose vitamin D versus placebo to prevent complications in COVID-19 patients: multicentre randomized controlled clinical trial. PLoS One. (2022) 17:e0267918. doi: 10.1371/journal.pone.0267918, PMID: 35622854 PMC9140264

[132] Bishop CW Ashfaq A Melnick JZ Vazquez-Escarpanter E Fialkow JA Strugnell SA. REsCue trial: randomized controlled clinical trial with extended-release calcifediol in symptomatic COVID-19 outpatients. Nutrition. (2023) 107:111899. doi: 10.1016/j.nut.2022.111899 36529089 PMC9639413

[133] Fernandes AL Murai IH Reis BZ Sales LP Santos MD Pinto AJ. Effect of a single high dose of vitamin D3 on cytokines, chemokines, and growth factor in patients with moderate to severe COVID-19. Am J Clin Nutr. (2022) 115:790–8. doi: 10.1093/ajcn/nqab426, PMID: 35020796 PMC8807215

[134] Murai IH Fernandes AL Sales LP Pinto AJ Goessler KF Duran CSC. Effect of a single high dose of vitamin D3 on hospital length of stay in patients with moderate to severe COVID-19: a randomized clinical trial. JAMA. (2021) 325:1053–60. doi: 10.1001/jama.2020.26848 33595634 PMC7890452

[135] Jolliffe DA Vivaldi G Chambers ES Cai W Li W Faustini SE. Vitamin D supplementation does not influence SARS-CoV-2 vaccine efficacy or immunogenicity: sub-studies nested within the CORONAVIT randomised controlled trial. Nutrients. (2022) 14:3821. doi: 10.3390/nu14183821 36145196 PMC9506404

[136] Mahjoub L Youssef R Yaakoubi H Salah HB Jaballah R Mejri M. Melatonin, vitamins and minerals supplements for the treatment of COVID-19 and COVID-like illness: a prospective, randomized, double-blind multicenter study. Explore. (2024) 20:95–100. doi: 10.1016/j.explore.2023.06.009, PMID: 37419768 PMC10281695

[137] Haas M Brandl B Schinhammer L Skurk T. Individualized supplementation of Immunoactive micronutrients and severity of upper respiratory infection symptoms-a randomized intervention study. Nutrients. (2024) 16:1400. doi: 10.3390/nu16101400, PMID: 38794638 PMC11123851

[138] Murai IH Fernandes AL Antonangelo L Gualano B Pereira RMR. Effect of a single high-dose vitamin D3 on the length of hospital stay of severely 25-Hydroxyvitamin D-deficient patients with COVID-19. Clinics. (2021) 76:e3549. doi: 10.6061/clinics/2021/e3549 34852148 PMC8595591

[139] Caballero-García A Pérez-Valdecantos D Guallar P Caballero-Castillo A Roche E Noriega DC. Effect of vitamin D supplementation on muscle status in old patients recovering from COVID-19 infection. Medicina. (2021) 57:1079. doi: 10.3390/medicina57101079 34684116 PMC8537350

[140] Brunvoll SH Nygaard AB Ellingjord-Dale M Holland P Istre MS Kalleberg KT. Prevention of covid-19 and other acute respiratory infections with cod liver oil supplementation, a low dose vitamin D supplement: quadruple blinded, randomised placebo controlled trial. BMJ. (2022) 378:e071245. doi: 10.1136/bmj-2022-071245 36215222 PMC9449357

[141] Cannata-Andía JB Díaz-Sottolano A Fernández P Palomo-Antequera C Herrero-Puente P Mouzo R. A single-oral bolus of 100,000 IU of cholecalciferol at hospital admission did not improve outcomes in the COVID-19 disease: the COVID-VIT-D-a randomised multicentre international clinical trial. BMC Med. (2022) 20:83. doi: 10.1186/s12916-022-02290-8 35177066 PMC8853840

[142] Villasis-Keever MA López-Alarcón MG Miranda-Novales G Zurita-Cruz JN Barrada-Vázquez AS González-Ibarra J. Efficacy and safety of vitamin D supplementation to prevent COVID-19 in frontline healthcare workers. A randomized clinical trial. Arch Med Res. (2022) 53:423–30. doi: 10.1016/j.arcmed.2022.04.003 35487792 PMC9013626

[143] Karonova TL Golovatyuk KA Kudryavtsev IV Chernikova AT Mikhaylova AA Aquino AD. Effect of cholecalciferol supplementation on the clinical features and inflammatory markers in hospitalized COVID-19 patients: a randomized, open-label. Single-Center Study Nutr. (2022) 14:2602. doi: 10.3390/nu14132602 PMC9268385 35807783

[144] De Niet S Trémège M Coffiner M Rousseau AF Calmes D Frix AN. Positive effects of vitamin D supplementation in patients hospitalized for COVID-19: a randomized, double-blind. Placebo-Controlled Trial Nutr. (2022) 14:3048. doi: 10.3390/nu14153048 PMC9330587 35893907

[145] van Helmond N Brobyn TL LaRiccia PJ Cafaro T Hunter K Roy S. Vitamin D3 supplementation at 5000 IU daily for the prevention of influenza-like illness in healthcare workers: a pragmatic randomized clinical trial. Nutrients. (2022) 15:180. doi: 10.3390/nu15010180, PMID: 36615837 PMC9823308

[146] Elamir YM Amir H Lim S Rana YP Lopez CG Feliciano NV. A randomized pilot study using calcitriol in hospitalized COVID-19 patients. Bone. (2022) 154:116175. doi: 10.1016/j.bone.2021.116175 34508882 PMC8425676

[147] Dilokpattanamongkol P Yan C Jayanama K Ngamjanyaporn P Sungkanuparph S Rotjanapan P. Impact of vitamin D supplementation on the clinical outcomes of COVID-19 pneumonia patients: a single-center randomized controlled trial. BMC Complement. Med. Ther. (2024) 24:97. doi: 10.1186/s12906-024-04393-6, PMID: 38383361 PMC10880207

[148] Maghbooli Z Sahraian MA Jamalimoghadamsiahkali S Asadi A Zarei A Zendehdel A. Treatment with 25-Hydroxyvitamin D(3) (Calcifediol) is associated with a reduction in the blood neutrophil-to-lymphocyte ratio marker of disease severity in hospitalized patients with COVID-19: a pilot multicenter, randomized, placebo-controlled, double-blinded clinical trial. Endocr Pract. (2021) 27:1242–51. doi: 10.1016/j.eprac.2021.09.016, PMID: 34653608 PMC8511889

[149] Reino-Gelardo S Palop-Cervera M Aparisi-Valero N Espinosa-San MI Lozano-Rodríguez N Llop-Furquet G. Effect of an immune-boosting, antioxidant and anti-inflammatory food supplement in hospitalized COVID-19 patients: a prospective randomized pilot study. Nutrients. (2023) 15:1736. doi: 10.3390/nu15071736, PMID: 37049576 PMC10096722

[150] Tosi N Favari C Bresciani L Flanagan E Hornberger M Narbad A. Unravelling phenolic metabotypes in the frame of the COMBAT study, a randomized, controlled trial with cranberry supplementation. Food Res Int. (2023) 172:113187. doi: 10.1016/j.foodres.2023.113187 37689939

[151] Lackner S Mahnert A Moissl-Eichinger C Madl T Habisch H Meier-Allard N. Interindividual differences in aronia juice tolerability linked to gut microbiome and metabolome changes-secondary analysis of a randomized placebo-controlled parallel intervention trial. Microbiome. (2024) 12:49. doi: 10.1186/s40168-024-01774-4, PMID: 38461313 PMC10924357

